# Dummy-atom modelling of stacked and helical nanostructures from solution scattering data

**DOI:** 10.1107/S2052252518005493

**Published:** 2018-05-11

**Authors:** Max Burian, Heinz Amenitsch

**Affiliations:** a Institute of Inorganic Chemistry, Graz University of Technology, Stremayrgasse 9/V, Graz 8010, Austria

**Keywords:** SAXS, stacked structures, helical structures, shape retrieval, *SasHel*, structure determination, solution scattering, computational modelling, structural biology, nanoscience

## Abstract

The availability of dummy-atom modelling programs to determine the shape of monodisperse globular particles from small-angle solution scattering data has led to outstanding scientific advances. However, there is no equivalent procedure that allows modelling of stacked, seemingly endless structures, such as helical systems. This work presents a bead-modelling algorithm that reconstructs the structural motif of helical and rod-like systems. The algorithm is based on a ‘projection scheme’: by exploiting the recurrent nature of stacked systems, such as helices, the full structure is reduced to a single building-block motif. This building block is fitted by allowing random dummy-atom movements without an underlying grid. The proposed method is verified using a variety of analytical models, and examples are presented of successful shape reconstruction from experimental data sets. To make the algorithm available to the scientific community, it is implemented in a graphical computer program that encourages user interaction during the fitting process and also includes an option for shape reconstruction of globular particles.

## Abstract 2

A bead-modelling approach is presented to determine the structural motifs of helical and rod-like systems from small-angle solution scattering data. The implemented algorithm is verified using analytical models and is further applied to reconstruct from experimental scattering data the building block of a self-assembled peptide double helix.

## 1. Introduction   

Small-angle X-ray scattering (SAXS) is an established technique to study the structural aspects, such as the size and shape, of molecular systems in solution (Li *et al.*, 2016 ▸). As this structural information is not directly apparent from the recorded scattering intensity, one requires a fitting process that generally relies on an underlying mathematical model (Pedersen, 1997 ▸). While a variety of such analytical models are available in the literature (Pedersen, 1997 ▸), each of them is bound to a given shape. The fitting process of an experimental data set therefore requires previous knowledge of the sample such that the appropriate model can be chosen. Dummy-atom (DA) modelling, which describes the particle shape as a variable bead assembly, bypasses this issue as the fitting process is no longer constrained to a single geometry (Chacón *et al.*, 1998 ▸; Walther *et al.*, 2000 ▸; Svergun *et al.*, 2001 ▸; Franke & Svergun, 2009 ▸; Koutsioubas *et al.*, 2016 ▸). Consequently, no *a priori* knowledge regarding the solute’s shape is necessary. This advancement has helped to make SAXS an attractive technique to characterize stable molecules in solution, far beyond the community of scattering experts (Yang, 2014 ▸).

However, single molecules in solution are not always stable. In fact, it is in their nature to interact and aggregate, often resulting in highly organized hierarchical systems stretching over several orders of magnitude (Palmer & Stupp, 2008 ▸; Wasielewski, 2009 ▸; Busseron *et al.*, 2013 ▸; Praetorius & Dietz, 2017 ▸). Helical and chiral superstructures are a common yet spectacular class of such systems, *e.g.* the length of the human genome DNA double helix exceeds several centimetres (Venter *et al.*, 2001 ▸) while the forces that give rise to the characteristic helical motif act on the molecular (nanometre) level (Dobbs, 2007 ▸). Amyloid fibrillation, *i.e.* the aggregation of proteins into insoluble, often helical, fibrils, has been linked to critical diseases such as type 2 diabetes or Alzheimer’s (Dobson, 2003 ▸; von Bergen *et al.*, 2000 ▸) as well as to unwanted drug degradation (Morozova-Roche & Malisauskas, 2007 ▸; Vestergaard *et al.*, 2007 ▸). Consequently, a comprehensive understanding of these systems requires structural characterization at the (supra-)molecular scale. Small-angle X-ray diffraction of aligned fibres [*e.g.* the famous studies of the structure of DNA (Wilkins *et al.*, 1953 ▸; Watson & Crick, 1953 ▸)] is a powerful technique for this purpose. However, the evaluation of solution scattering data from randomly oriented helical structures faces two challenges: (i) only a few analytical models are available from which structural information, such as pitch and twist, can be retrieved (Schmidt, 1970 ▸; Pringle & Schmidt, 1971 ▸; Hamley, 2008 ▸; Teixeira *et al.*, 2010 ▸); and (ii) the endless nature of helices does not allow DA modelling using current programs (Volkov & Svergun, 2003 ▸; Gingras *et al.*, 2008 ▸).

In this work, we present a bead-modelling algorithm to determine the structural motif of monodisperse systems, highly elongated in one dimension, in solution from SAXS data. We use symmetrical boundary conditions to project the seemingly infinite nature of *e.g.* helical systems onto a single building-block unit, represented by dummy atoms (DA). This building block is altered by random DA movements while simultaneously fitting the corresponding scattering curve against the experimental one. The proposed method is verified using simulated data sets of various one-dimensional structures and we subsequently apply it to experimentally obtained SAXS data. The full algorithm is implemented in a computer program (*SasHel*), which also includes an option for globular geometries. The reduction of the system’s complexity by symmetric projection and the fast code implementation result in a toolkit that allows a full shape retrieval from scattering data in the order of 3–90 min on a standard work station (see Appendix *A* [App appa]), depending on the level of detail.

## 2. Projection scheme   

Dummy-atom modelling is based on the idea that a given particle shape is represented by an assembly of *N* small beads of equal scattering length density, called dummy atoms (DA). According to the Debye formula (Debye, 1915 ▸), the scattering intensity of this assembly can then be calculated from the bead assembly as 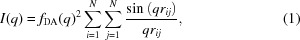
where the norm of the scattering vector is denoted as *q* = 4πsin(θ)/λ (2θ is the scattering angle and λ is the radiation wavelength) and *f*_DA_(*q*) represents the DA form factor (given in this work by a Gaussian sphere approximation; Koutsioubas & Pérez, 2013 ▸; Svergun *et al.*, 1995 ▸; Grudinin *et al.*, 2017 ▸; Fraser *et al.*, 1978 ▸). A DA diameter of 0.2 nm was used for all reconstructions. As this formalism considers the distances between all possible DA pairs *r_ij_* = |**r**_*j*_ − **r**_*i*_| inside the assembly, its mathematical complexity is 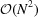. Hence, the computational effort increases drastically for large *N*. Adequate modelling of seemingly infinite rod-like geometries requires very large numbers of DAs (*N* 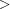 10 000), making the Debye formula appear inadequate in its standard notation.

**Figure 1:**
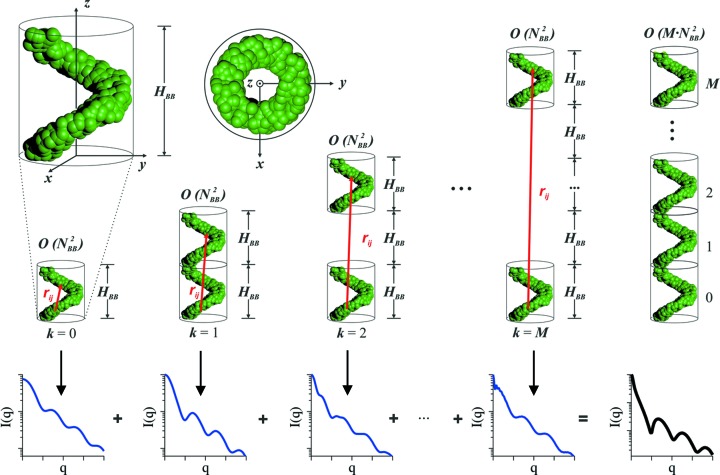
The projection scheme, visualized using the example of a single-stranded helix. For such seemingly endless geometries, there is a building block (upper left-hand corner) that is recurrent along the *z* axis (see full helix on the right). For a full body consisting of *M* stacked building blocks, one finds distinct structural motifs, such as the building block itself, neighbouring building blocks, single-spaced building blocks and so on up to (*M* − 1)-spaced building blocks. The scattering intensity of the full geometry can hence be calculated by summing the contributions of these structural motifs, scaled by their recurrence. This bypasses the demand for an actual DA model representing the full structure, as we solely evaluate projections of the building block instead of actual stacked duplicates, hence the term projection scheme.

Rod-like structures, such as helices, often possess a certain structural motif, a building block, which recurs along the elongation direction. Hence, only this building block is of interest for shape reconstruction. We define a building block of *N*_BB_ dummy atoms with its elongation direction parallel to the *z* axis (see Fig. 1 ▸). The full rod-like structure is then reproduced by duplicating the building block *M* times along the *z* direction, with a stacking distance corresponding to the building block’s height *H*_BB_ (see Fig. 1 ▸). The evaluation of this stacked model by means of the Debye formula [equation (1)] now includes redundant terms, as distinct motifs inside the structure are now recurrent. For instance, the single building-block motif would be evaluated at each repetition along **z**, such that the same computation is performed a total of *M* times. It is therefore sufficient to calculate the scattering intensity of the building block only once and scale it by the number of stacks. The same principle applies to inter-building-block contributions. For example, as the motif of neighbouring building blocks can be found (*M* − 1) times in the full structure, we can evaluate this motif once and, again, multiply it by the corresponding scalar.

In mathematical terms, the Debye formula can be adjusted to neglect these redundancies, resulting in 
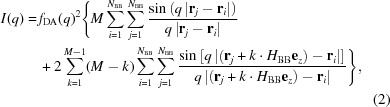
where **e**_*z*_ denotes the unit vector along the *z* axis (elongation/stacking direction). This formalism reduces the calculation of the scattering intensity to the sum of structural motifs found inside the stacked model (see scheme in Fig. 1 ▸). It further bypasses the demand for a DA model to represent the full structure, as we evaluate only projections of the building block instead of all stacked duplicates, hence the term ‘projection scheme’. Its benefit, compared with the standard Debye formula, is a reduction in the mathematical complexity from 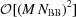 to 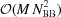. In the case of the example shown in Fig. 1 ▸ (*N*_BB_ = 400, *M* = 15), this increases the calculation speed approximately seven fold.

The notion of reducing a given model to its structural motifs raises a critical point regarding the required number of stacks *M* necessary to represent a seemingly endless structure. This number defines the length of the entire projected model *L*, simply *via* *L* = *M* *H*_BB_. According to scattering theory, rod-like structures present a characteristic *q* ^−1^ power-law behaviour in the intermediate Porod regime (Glatter & Kratky, 1982 ▸), whereas the transition from this Porod regime (*q* ^−1^) to the adjacent lower-angle Guinier regime (*q* ^−0^) occurs at a specific scattering vector *q*_1_ depending on the length of the rod, which can be estimated using *q*_1_ = 18^1/2^/*L* in the simplified framework of the Hammouda model (Hammouda, 2010 ▸). Thus, if the experimental data present a *q* ^−1^ power law at even the smallest accessible scattering angle *q*_min_, the length of the structure must be larger than *L* 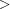 18^1/2^/*q*_min_. Similarly, we use this relation to determine the minimum number of stacks required, according to *M* 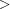 2 + 18^1/2^/(*H*_BB_ *q*_min_) (with the additional term of + 2 to avoid truncation effects).

## 3. Determination of the stacking distance   

The projection scheme is a faster alternative for calculating the scattering intensity of a stacked structure compared with the standard Debye formula. As is apparent from equation (2), the formalism requires an additional input parameter, the stacking distance between the building blocks *H*_BB_. In the following, we present a pathway to determine this using the pair distance distribution function (PDDF).

The PDDF corresponds to the surface-weighted probability to find two points separated by a distance *r* inside a particle (Glatter & Kratky, 1982 ▸). It is thus a histogram of all distances that appear inside a given particle, weighted by the corresponding electron densities. In the case of DA assemblies, the PDDF can be also interpreted as the volume-weighted pair correlation function *p*(*r*).

An analytically available example of this definition yields the case of stacked spheres using the formalism based on the work of Glatter (1980 ▸), from which a series of conclusions can be drawn (see Fig. S1 in the supporting information for a system of ten stacked spheres). Evidently, as the system contains ten spheres the corresponding PDDF presents ten peaks. The first peak (blue trace in Fig. S1) relates to the mean shape of all the spheres involved – it is hence an intra-building-block PDDF. The following nine peaks (red traces in Fig. S1) are caused by the repetitive nature of the system, as for each possible sphere-to-sphere distance a new peak is found – they are hence inter-building-block PDDFs. These inter-building-block PDDFs, in particular the distances between them, therefore hold information on the stacking distance between the building blocks.

In order to determine the stacking distance of a structure from a given PDDF, it would be intuitive to measure the peak distances. However, the exact peak shapes and therefore positions are distorted due to (i) the linear high-*r* decay in the PDDF (see dashed lines in Figs. S1 and S2) and (ii) the overlap of neighbouring peak contributions (Glatter, 1980 ▸; Feigin & Svergun, 1987 ▸). A direct measurement of the peak positions in the PDDF might therefore result in misdetermination of the stacking distance. A stable approach to circumvent this issue is to calculate the derivative of the PDDF (dPDDF). This numerically fast and easy operation suppresses the above-mentioned decay distortion (see dPDDF in Fig. S1). The resulting dPDDF can then be fitted by *e.g.* a damped sinusoidal function in which the period is directly related to the mean stacking distance (see Fig. S1 and Section S1 in the supporting information).

A more complex example illustrating the named distortion effects is the case of a torus (a ring with a circular cross section; Kawaguchi, 2001 ▸). Such a torus presents two characteristic dimensions: the ring–centre diameter and the ring thickness. As a result, the PDDF of such a single torus exhibits two distinct peaks correlating to these structural features (see Fig. S2; the model scattering data, including an artificial error band, were calculated according to Appendix *A* [App appa]). Considering now the case of multiple stacked rings (Kornmueller *et al.*, 2015 ▸), for each ring that is added on top of the other(s) we find a new peak in the corresponding PDDF (see Figs. S2 and S3). However, in this case, the radial size of the tori (diameter 20 nm) was selected to be larger than the stacking distance between them (7 nm), resulting in an increased distortion of the inter-building-block peak positions [see distortion phenomenon (ii) above, as well as Fig. S2]. As shown in Fig. S2, the determination of the stacking distance by fitting of the dPDDF yields a stable result.

We employ this approach to determine the stacking distance from all repetitive structures presented in this work. The advantage of this choice is that we avoid the use of a complementary characterization technique, as the underlying PDDF can be determined from a given data set using a variety of available software packages. A more detailed discussion of the limitations and possible causes of misdetermination can be found in Sections S1.2 and S1.3 and Figs. S1–S6.

## 4. Fitting algorithm   

According to the presented projection scheme, we reduce a seemingly infinite structure to a building-block motif together with two boundary conditions, the stacking distance *H*_BB_ and the number of necessary stacks *M*. As these two values are determined directly from either the PDDF or the scattering data, the modelling occurs within the single building-block volume. We thus represent the building-block motif by a dummy-atom configuration *X* such that the scattering intensity *I*_calc_(*q*_m_) (where *q*_m_ denotes the *q* value of the measured data points) can be calculated using equation (2). To find a configuration that best fits the experimental scattering intensity *I*_exp_(*q*_m_), the relation 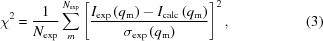
needs to be minimized, where σ_exp_(*q*_m_) denotes the experimental error and *N*_exp_ the number of data points.

In DA modelling, the principle idea behind this minimization procedure is straightforward: a given initial configuration is gradually altered until the best agreement between the corresponding scattering curves is found. Here, we randomly fill an initial building-block volume with DAs that are set free at the start of the fitting procedure (500–1500 DAs per building block are suggested, depending on the experimental resolution – see Section S2). In order to optimize the target function χ^2^, various metaheuristic methods exist such as Simulated Annealing (Kirkpatrick *et al.*, 1983 ▸) or the Genetic Algorithm (Mitchell, 1996 ▸). In our case, we employ a metaheuristic fitting procedure (Boussaïd *et al.*, 2013 ▸; Gogna & Tayal, 2013 ▸) with an antifragile implementation (Taleb & Douady, 2013 ▸) and the algorithm improves χ^2^ by randomly moving single DAs. In contrast with current DA modelling programs, the DAs do not move on an underlying grid. As the magnitude of the movement is scaled by a temperature factor, we force the system to freeze eventually in a given condition and the algorithm to converge.

DA modelling faces the general problem of uniqueness: as models consist of >10^3^ DAs, the information content given by the scattering data is highly overdetermined by a given configuration. Fitting of the scattering data without restraints can therefore lead to physically unfeasible results, in particular with regard to the model homogeneity and compactness. Current algorithms control and optimize these two properties during the fitting process by means of a ‘looseness penalty’ regularization term as a quantitative measure of the DAs’ local vicinity: by counting and maximizing the number of contacting neighbours of each DA, a compact and homogeneous configuration is achieved (Svergun, 1999 ▸; Franke & Svergun, 2009 ▸; Koutsioubas *et al.*, 2016 ▸). We adapt this proven technique to our grid-free algorithm in two ways. First, we introduce the parameter *d*_N12_, denoting the distance between a given DA and its 12 (close-packed limit) nearest neighbours. Similar to the looseness penalty, *d*_N12_ quantifies the local vicinity of each DA, acting as a homogeneity classifier for the algorithm to decide if a given DA position is accepted or not. Second, we apply the idea of a regularization term and introduce a ‘radial compactness’ parameter RC(*X*) into the minimization procedure, keeping the DA close to the (radial) centre of mass of the configuration.

As the choice of a grid-free DA algorithm is a departure from current implementations, we face challenges for which no readily available solutions exist. In particular, we must introduce new concepts, (i) to obtain a hard-contact limit for neighbouring DAs, (ii) to allow model scalability over different length scales and (iii) to generate a random movement depending on the current annealing temperature. In the following subsections we discuss these topics in more detail, starting with the introduction of the scaling parameter *D_X_* and the random-movement generator, and then moving to the mathematical definitions of *d*_N12_ and RC(*X*) regarding homogeneity and compactness. A general overview of these concepts and their parameters is given in Table 1 ▸. At the end of this section, we further address the topic of model uniqueness and repeatability and give a conclusive overview of the algorithm’s implementation in the program *SasHel*.

### 4.1. Scalability – scaling parameter *D_X_*   

Allowing DAs to move randomly without an underlying grid brings an advantage in terms of the model volume and therefore the radial size. In a fixed grid system, the final DA density is predefined by the grid resolution, which is related to the resolution of the scattering pattern in reciprocal space. In the case of reconstruction of a highly elongated structure, radial expansion of the DA configuration during the fitting process is only possible by the addition of new DAs, hence increasing the numerical overhead (*N*_DA_ ≃ 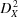 for radial growth). Radial compression, on the other hand, is limited by the grid resolution, such that at a certain point the grid type (*e.g.* hexagonal packing of the DAs) may impose artificial structural features. This is particularly true for the case of highly elongated structures, where the grid resolution is likely to be more coarse than the experimental limit as a reasonable computational overhead (*N*_DA_ < 10 000) must be maintained. Our grid-free approach does not present such limitations: as the number of DAs per building block (mainly determined by the experimental resolution) is kept constant, the algorithm on its own adapts to a change in DA density while at the same time maintaining the relative model resolution and computational resources (see Section S2.2 for a discussion of model resolution). This in particular benefits users who have chosen the wrong radial size for the starting configuration, as the algorithm adapts according to the scattering curve without computational drawbacks. We exploit this adaptive potential of our algorithm using an estimated diameter *D_X_* of a given configuration *X*, which acts as a scaling parameter throughout the algorithm.

We quantitatively track the growing or shrinking of the DA configuration *X* throughout the fitting process *via* the radius of gyration *R*_G,*X*_. For elongated cases, we assume a cylindrical reference geometry, such that we estimate *D_X_* after every DA movement according to 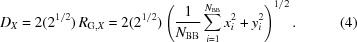
Along the *z* axis we introduce a continuity condition (see the limited building-block height in Fig. 1 ▸) such that DAs leaving the building block in the vertical direction are re-projected back inside the building-block volume.

### 4.2. Random-movement generator   

As we pursue the concept of grid-free random DA movements to find an optimal configuration, we require a random-movement generator that depends on a series of parameters, including (i) the dimensions of the current DA configuration (*H*_BB_ and *D_X_*), (ii) the current temperature *T* of the fitting process (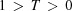) and (iii) the helical chirality of the sample (helical field bias γ). In every numerical iteration throughout the fitting process, a chosen DA is moved along the unit axes **e**_*x*_, **e**_*y*_ and **e**_*z*_ by the random vector **r**_rand_(*T*), where 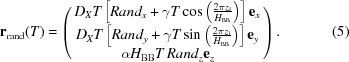
Here, a random number generator *Rand* returns any value between −1 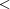 *Rand_x,y,z_* 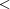 1 every time it is called. In order to adjust the random movement by the size of the current configuration, the radial and longitudal contributions are scaled by the current diameter *D_X_* or the building-block height *H*_BB_, respectively (movement along the *z* direction is scaled by α to ensure axial homogeneity, with a standard value of α = 0.1). Further, each movement along the *x*, *y* or *z* direction is scaled by the current temperature *T*. In the case of radial movement along the *x* or *y* direction, we added a helical bias term (depending on the DA’s current **e**_*z*_ position *z_i_*; the building-block motif extends from *z* = 0 to *z* = *H*_BB_). This bias term is scaled by the helical field bias γ (user defined with values of the order of 0 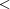 γ 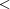 1, default value 0.3) as well as by the current temperature *T*, such that it favours counterclockwise solutions motivated by the first structural model of DNA (Watson & Crick, 1953 ▸). It is important to note that, mathematically, the helical bias term correlates quadratically with the current temperature *T*. Therefore, its contribution is only significant in the early stages of the fitting process (see Fig. S11 for its influence on the reconstruction) such that, using its default value of 0.3, it does not influence the modelling of non-helical systems (Fig. S15) but facilitates the reconstruction of high-aspect helical motifs.

In the case of fitting globular structures (*M* = 1), the random-movement vector **r**_rand_(*T*) is significantly simplified according to 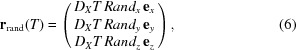
where the current diameter *D_X_* is now evaluated over all three directions, such that equation (4) now also includes the contribution of the DAs’ current *z* positions 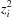.

### 4.3. Homogeneity – the *d*_N12_ parameter   

The random nature of DA movements causes a side effect with regard to the uniqueness of the fitted model: as there are infinite possibilities for describing a given structure by randomly filling it with point scatterers, the terminology of a unique model is not reasonable in this context. However, certain criteria related to the homogeneity of the DA configuration make a fitted DA reconstruction, in a physical sense, very unlikely. These scenarios are (i) high-density DA clusters and (ii) single disconnected DAs far away from the remaining configuration.

We optimize the homogeneity throughout the fitting process by analogy with the established looseness parameter in fixed-grid systems (Svergun, 1999 ▸; Franke & Svergun, 2009 ▸; Koutsioubas & Pérez, 2013 ▸), namely by evaluating the local vicinity around each DA. In these fixed-grid implementations, the distance between neighbouring DAs is known such that only the number of contacts needs to be counted. In our grid-free case, we use the same principle in an inverted manner: we assume an ideal close-packed condition with 12 neighbours and calculate their mean distance to the central DA, resulting in the parameter *d*_N12_. In the extreme case of a DA within a high-density cluster [scenario (i) above], *d*_N12_ will be very small. On the other hand, for a single free-floating DA that is far away from the core DA assembly [scenario (ii)], *d*_N12_ will be very large. The magnitude of *d*_N12_ is hence inversely proportional to the DA density around a given DA. Equally, the average *d*_N12_ over the full DA configuration *d*_N12,*X*_ denotes an inverse measure of the mean DA density of the configuration.

We use the *d*_N12_ parameter for a twofold purpose. In order to avoid scenario (i), we do not allow DAs to come closer than 0.1〈*d*_N12,*X*_〉. This acts as a hard-contact limit so that we circumvent DA clustering and therefore unfeasible singularities throughout the fitting process. In order to avoid scenario (ii), we repeatedly force DAs with *d*_N12_ 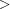 2〈*d*_N12,*X*_〉 that are outside the mean radial distance 〈|**r**|〉_*X*_ to move towards the centre of mass of the closest fraction of DAs. This avoids free floating of a single DA and therefore forces the DAs to remain in a compact configuration. A detailed description of how the *d*_N12_ parameter is implemented in the fitting algorithm can be found in Section S3 and Fig. S27.

### 4.4. Compactness – the radial compactness parameter RC(*X*)   

In order to prevent unphysical disassembly of the DA configuration during the fitting procedure, we retain the DAs close to the radial centre of mass (RCOM) of a given configuration. We achieve this by introducing a potential well such that the DAs are weighted as a function of their radial distance from the centre of mass 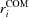. DAs inside the potential well should be unaffected, so that they move freely regardless of their position. Once DAs drift towards the well border, a force should push them back towards the centre of mass, hence avoiding radial disassembly. To find an adequate mathematical description of this potential field φ we specified the following criteria: (i) radial continuity to avoid non­linearities; (ii) asymptotic behaviour, such that 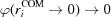 and 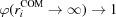; (iii) scalability in magnitude (0 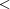 φ 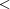 1); and (iv) scalable potential-well size and steepness. Based on these requirements (and inspired by the potential field for a Gaussian distribution of electrostatic charges; Schlick, 2010 ▸), we use the mathematical properties of the error function 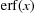 to define a radial-symmetric potential well acting on each DA. As a result, we obtain the radial compactness parameter RC(*X*) according to 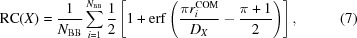
where 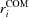 represents the radial distance between the RCOM of configuration *X* and the *i*th DA (in the case of globular geometries, 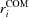 represents the distance between the centre of mass of configuration *X* and the *i*th DA). With regard to the specifications defined above, this formalism fulfils the above-mentioned points (i)–(iv) by means of the scaling parameter *D_X_* (which is evaluated after every DA movement). In particular, for point (iv), scalable potential-well size and steepness, the potential-well boundary (half-height position) will be found at 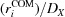 = (π +1)/2π = 0.66, where its derivative is 1/π. In absolute terms, for a very compact structure such as a cylinder with a smooth surface or an infinitely thin cylindrical shell, RC(*X*) will be approximately 0.06 or 0.24, respectively. Each single atom moving further away from *D_X_* will cause RC(*X*) to increase towards 1.

We account for this radial compactness throughout the fitting process by using RC(*X*) scaled by the compactness weight β as a regularization term. Thus, the algorithm in fact minimizes the function 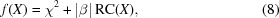
instead of only χ^2^ alone.

As an ultimate radial boundary, similar to the search-volume diameter in *DAMMIN* (Svergun, 1999 ▸), we apply a critical diameter *D*_*X*,crit_ = 2*D_X_* such that single DAs moving far away from the RCOM are repeatedly forced into a more compact configuration (see Section S3 and Fig. S27 for a detailed description of how the radial compactness is implemented in the algorithm).

### 4.5. Model resolution and uniqueness   

The most obvious visual side effect of using random movements is related to the outer surface of the fitted model. For example, if DAs can only move on an artificial grid, the fitted configuration of a globular particle will present a surface smoothness according to the lattice planes of the grid. As already discussed above, allowing random DA movements results in infinite possibilities for representing a given particle volume and thus also its surface. Consequently, the surface of a random-movement fitted configuration will be significantly rougher than a corresponding fixed-grid model. However, this increased surface roughness is just a visual symptom of the information content provided by the scattering curve, as we discuss in the following.

In absolute terms, we can expect to end up with a fitted configuration that presents structural features, both on the surface and within the volume, that are below the resolution limit of the experimental data (*d*_min_ = π/*q*_max_, where *q*_max_ is the upper angular range of the experimental data). This implies that *e.g.* thin helical tapes require a large accessible angular range in order to be resolved properly. If this is not the case, the retrieved model is at high risk of being over-interpreted. On a less obvious note, a low angular resolution can further lead to artefacts within the configuration that might not be seen by a common surface representation (we address this topic in more detail in Section S2).

A common technique to avoid misinterpretation of such artefacts is to test the reproducibility of the reconstruction (Volkov & Svergun, 2003 ▸). This approach brings a series of advantages. First, the overall stability and reliability of the reconstruction from a given data set are assessed. Second, a consecutive averaging process of all reconstructions projects the DAs onto an artificial occupancy-weighted grid, which provides a straightforward procedure to determine DA validity and volume inhomogeneity. Third, this occupancy map helps to identify structural artefacts in single reconstructions caused by insufficient information content of the scattering data, thus avoiding over interpretation. Fourth, the reconstruction of an arbitrary shape from scattering data is a (highly) underdetermined problem so it is not guaranteed to obtain a unique result from a given fitting algorithm. Performing a reproducibility analysis when reconstructing a structural motif from experimental scattering data, in particular when the information content and validity of the data are questionable, is hence highly recommended. In quantitative terms, the reproducibility of a reconstruction may be judged by the mean normalized spatial discrepancy 〈NSD〉 of parallel reconstructions, which represents an measure of dissimilarity (〈NSD〉 = 0 for identical models) for the independent runs (Volkov & Svergun, 2003 ▸).

### 4.6. Implementation   

We implemented the fitting algorithm in the computer program *SasHel*, a *Qt* graphical user interface (GUI) written in C++ that allows user interaction. When starting a fitting procedure, the program generates a random cylindrical starting configuration according to a user-defined diameter, the building-block stacking distance and the number of DAs per building block. Once started, the fitting algorithm undergoes *N_k_* iterations: starting from a temperature *T*_0_, the system cools down as defined by the quenching coefficient *q_T_* (0 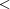 *q_T_* 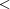 1) such that the current temperature at a given iteration *k* is *T_k_* = 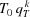 (we recommend the default values *N_k_* = 100, *T*_0_ = 1 and *q_T_* = 0.99 as an initial set of parameters for convergence). In each *k* iteration, all the DAs are randomly moved using the random-movement generator (see Section 4.2) under the restraints explained in Section 4.3, such that a movement is only accepted if (i) the new DA position complies with the hard-contact limit 0.1〈*d*_N12,*X*_〉 (if not, up to 100 new movements are considered) and (ii) an improvement in the function *f*(*X*) is found [see equation (8)]. After each *k* iteration the sequence of DAs is randomly mixed to avoid sequential biasing of the algorithm.

Throughout the fitting procedure, we pursue the concept of antifragility (Taleb & Douady, 2013 ▸), a concept applicable to metaheuristic optimization (Boussaïd *et al.*, 2013 ▸; Gogna & Tayal, 2013 ▸): the converging system is repeatedly forced out of its local minimum such that a global minimum is more likely to be found. These non-optimal moves are forced onto the configuration without consideration of the damage caused to the model, making the algorithm less prone to distortion of the solution space by regularization terms. A summary of the full algorithm, including an example implementation in pseudo-code, can be found in Section S3 and Fig. S27.

The program *SasHel* further includes a ‘parallel mode’ that runs a unique reconstruction on each available CPU core, so the model’s validity and reproducibility can easily be tested using *e.g.* *DAMAVER* (Volkov & Svergun, 2003 ▸).

## 5. Model examples   

To test the implemented algorithm, we simulated a number of scattering intensities from seemingly endless bodies (see Appendix *A* [App appa] for detailed dimensions). All reconstructions were performed using the default fitting parameters as follows: starting temperature *T*_0_ = 1, quenching coefficient *q_T_* = 0.99, number of iterations *N_k_* = 200, helical bias parameter γ = 0.3, compactness weight β = 1, DA form factor diameter *D*_DA_ = 0.2 nm and number of stacked building blocks *M* = 10. The dimensions of the initial random configuration, including the stacking distance of the building blocks, were determined from the relative dPDDFs (see Figs. S12 and S13). For all reconstructions, we used 500–800 DAs per building block, resulting in computation times for each run between 20 and 60 min on a standard workstation, respectively.

**Figure 2:**
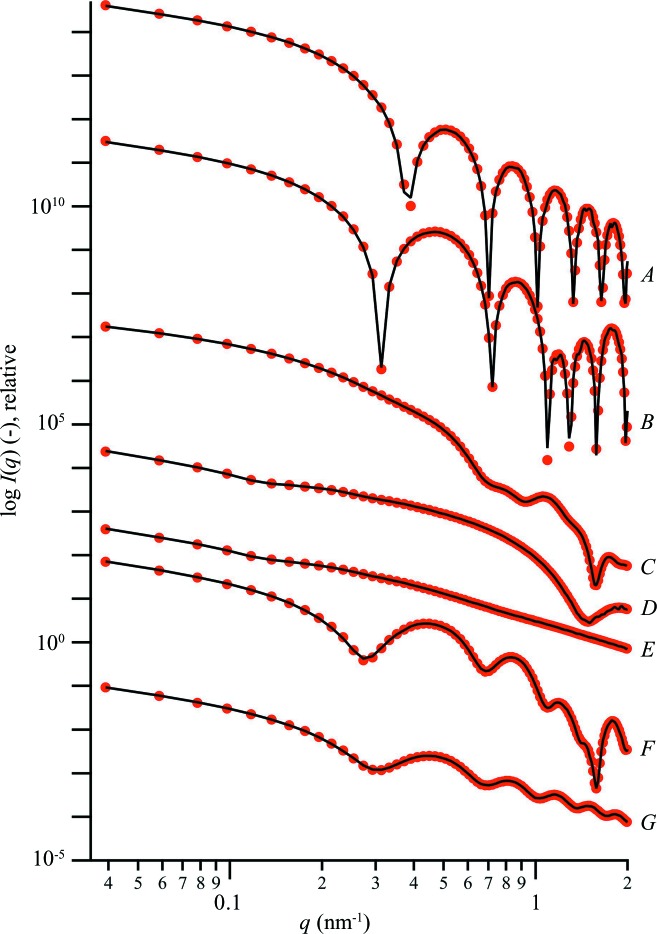
Scattering curves computed from the seemingly endless model geometries *A*–*G* shown in Fig. 3 ▸ (red circles; see Appendix *A* [App appa] for model details) and fits from the reconstruction (black lines). For better visualization, the scattering patterns are shifted vertically and error bars have been omitted (see Fig. S14 for high-*q* magnifications of these curves).

The scattering intensities of the theoretical models are shown in Fig. 2 ▸, along with the fits from the reconstructions. For all geometries used, we find agreement between the theoretical and fitted scattering intensities (the χ^2^ value of all fits is below the threshold of 0.1). Yet, cases *A*–*D* show slight oscillations in the higher-*q* regime 1 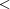 *q* 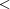 2 nm^−1^ (see Fig. S14), which is a resonance effect caused by the stacking nature of identical building blocks (see Section S2.3).

**Figure 3:**
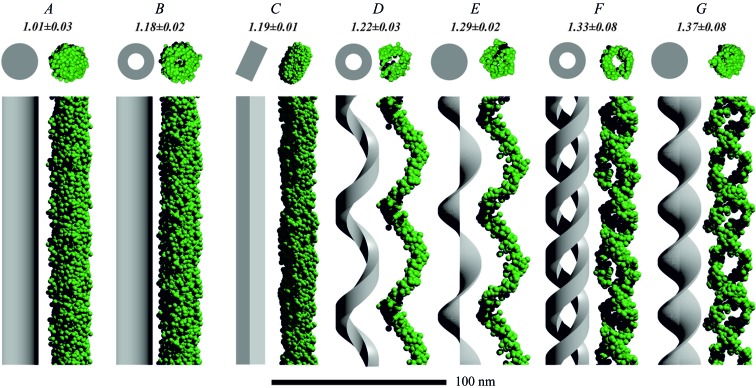
Theoretical and reconstructed three-dimensional models of the seemingly endless geometries used here. The numbers below the labels denote the mean normalized spatial discrepancy 〈NSD〉, obtained from eight independent reconstructions (random artificial cylinder: 〈NSD〉 = 1.03 ± 0.01, random artificial single-strand helix: 〈NSD〉 = 1.06 ± 0.01). The PDDFs and dPDDFs used to determine the stacking distance between the building blocks can be found in Figs. S12 and S13. See Fig. 2 ▸ for the corresponding scattering intensities and Fig. S15 for point representations, as denoted by the letters *A*–*G*.

Fig. 3 ▸ presents the corresponding three-dimensional models and reconstructions (see Fig. S15 for point representations). Models *A* and *B* show a circular cross section in agreement with the model, while in the case of the cylindrical shell the empty core is present. Further, the rectangular cross section of model *C* is visible in the reconstructed model. However, the sharp corners are not fully resolved, which is most likely the effect of insufficient resolution from the scattering data (*d*_res_ ≃ 1.6 nm compared with the rectangular cross section *a* × *b* = 4 × 20 nm). In cases *D*–*G*, the helical fingerprints (single- or double-stranded nature) are well resolved in the reconstructed models. Cases *D* and *E*, and *F* and *G*, present noticeable differences in their cross sections, helping to distinguish between a helical filament (empty core) and a helical tape (filled core). Further, variations in the thickness of the helical strands can be observed. The overall agreement between model and reconstruction for the main features demonstrates the functionality of the algorithm for similar seemingly endless bodies.

In order to evaluate the stability and reproducibility of the reconstruction algorithm, eight independent runs for each model geometry were analysed using *DAMAVER* according to the literature (Volkov & Svergun, 2003 ▸). Accordingly, we obtained the mean normalized spatial discrepancy 〈NSD〉 as a measure of the similarity of independent reconstructions of each model (〈NSD〉 = 0 for identical configurations). As shown in Fig. 3 ▸, the 〈NSD〉 of models *A*–*G* gradually increases from 1.01 to 1.37 with the complexity of the model. On an absolute scale, these 〈NSD〉 values are higher than those in the literature (Volkov & Svergun, 2003 ▸), where stable reconstructions were linked to an 〈NSD〉 of 0.4–0.7. The reason for this difference is found in the grid-free nature of our approach: NSD values for grid-free programs such as *GASBOR* are generally higher (Svergun *et al.*, 2001 ▸). To validate our findings, we constructed eight randomly filled artificial cylinders and single-strand helices (*N*_DA_ = 700), yielding 〈NSD〉 values of 1.03 ± 0.01 and 1.06 ± 0.01, respectively. These values, in context with the 〈NSD〉 of the above reconstructions, provide a reference for grid-free models where an 〈NSD〉 between 1 and 1.4 is observed.

It is also possible to run the fitting algorithm using only *M* = 1 stacks, which corresponds to the case of the building block alone. Thus, the same program can also be used to fit globular particles. We tested this option in a similar way to the method outlined above by simulating a number of scattering intensities from globular bodies (see Appendix *A* [App appa] for detailed dimensions). For all reconstructions, we used the same default fitting parameters as mentioned previously. The dimensions of the initial random configurations were determined from the maximum dimension found in the relative PDDFs (see Fig. S12). However, we increased the number of DAs to 800–1200, now resulting in computation times between 5 and 10 min per run on a standard workstation, respectively.

**Figure 4:**
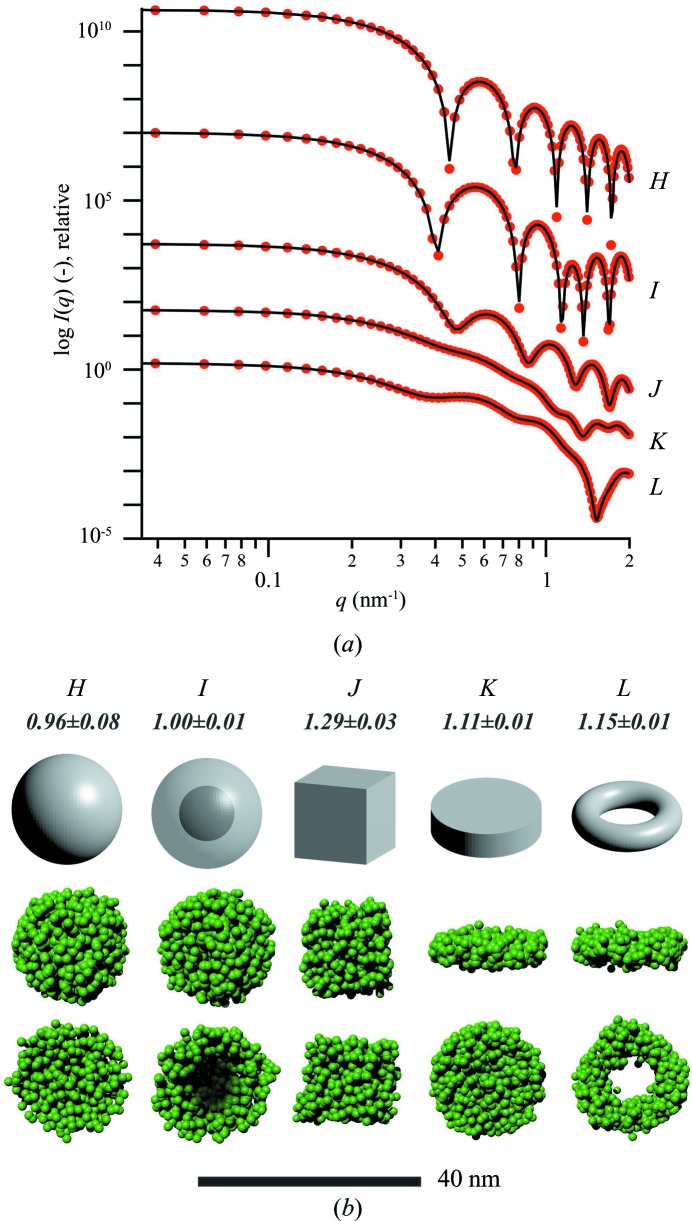
(*a*) Scattering curves computed from globular model geometries (red circles; see Appendix *A* [App appa] for model details) and fits from the reconstruction (black lines). For better visualization, the scattering patterns are shifted vertically and error bars have been omitted. The PDDFs of all models can be found in Fig. S12. (*b*) Theoretical and reconstructed three-dimensional models of the globular geometries used here (see Fig. S16 for point representations of the reconstructions). The numbers below the labels denote the mean normalized spatial discrepancy 〈NSD〉, obtained from eight independent reconstructions (random artificial sphere: 〈NSD〉 = 1.04 ± 0.01, random artificial cube: 〈NSD〉 = 1.06 ± 0.01).

The scattering intensities of the theoretical models and the fits from the reconstructions are shown in Fig. 4 ▸(*a*). For all geometries used, we find agreement between the theoretical and fitted scattering intensities (the χ^2^ value of all fits is below the threshold of 0.1). Evidently, the oscillations previously found for the repeating motif in the higher-angle regime (see Fig. 2 ▸) are not present. The three-dimensional models and reconstructions corresponding to the scattering patterns are shown in Fig. 4 ▸(*b*) (see Fig. S16 for point representations). Also in this case, the fitted morphologies clearly represent the corresponding models, including the hollow geometries *I* and *L*. The 〈NSD〉 values for eight independent reconstructions of each geometry are within the range 0.96–1.28, as denoted in Fig. 4 ▸(*b*) [in relative terms, the 〈NSD〉 values for eight randomly filled artificial spheres and cubes (*N*_BB_ = 1500) were 1.04 ± 0.01 and 1.06 ± 0.01, respectively]. These findings validate the use of the algorithm for globular bodies as well.

## 6. Experimental examples   

**Figure 5:**
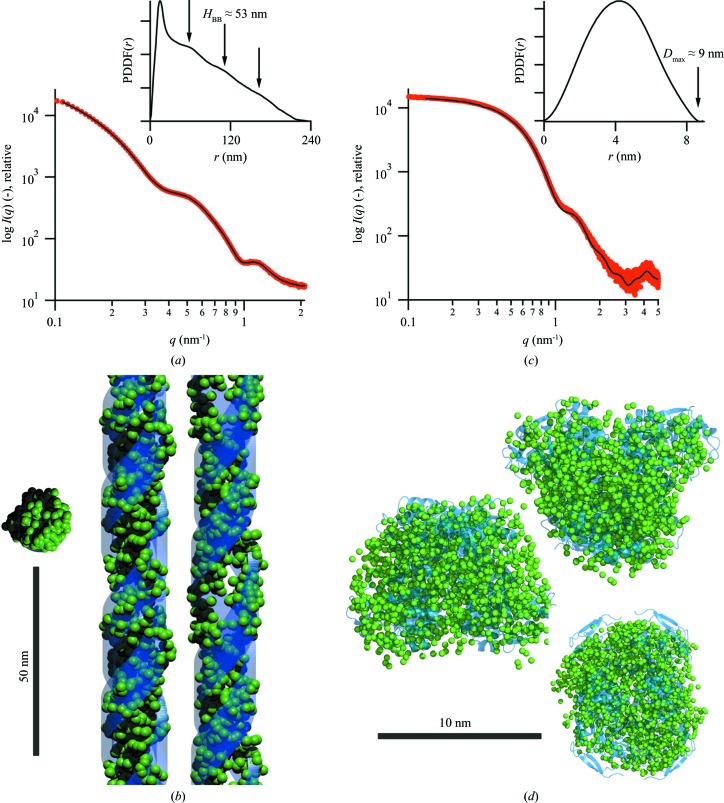
Verification of the reconstruction algorithm using experimental scattering data. (*a*) Experimental scattering data (error bars omitted for clarity) and fitted curve from the reconstruction of the self-assembled peptide double helix. The corresponding PDDF is shown in the inset, whereas the dPDDF used to determine the stacking distance can be found in Fig. S13. (*b*) Orthogonal views of the model reconstructed from the scattering pattern in panel (*a*) (green) compared with the previously published structure (blue). (*c*) Experimental data, fitted curve and PDDF for alcohol de­hydrogenase 1 (ADH). (*d*) Orthogonal views of the model reconstructed from the scattering pattern in panel (*c*) (green) compared with the crystal structure model (blue). See Fig. S17 for point representations of the reconstructions shown in panels (*b*) and (*d*), and Fig. S18 for a numerical stability analysis of both reconstructions.

As final example, we applied the described reconstruction procedure to experimental data for a self-assembled peptide double-strand helix (Kornmueller *et al.*, 2015 ▸). Prior to the fitting process, we determined a building-block stack spacing of 53 nm from the corresponding PDDF (see inset in Fig. 5 ▸ and Fig. S13). The scattering data were then fitted using 800 DAs over the angular range 0.08 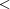 *q* 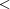 2.14 nm^−1^, resulting in a real-space resolution of approximately π/*q*_max_ = 1.5 nm, where the default fitting parameters according to Section 4 were used.

As shown in Fig. 5 ▸(*a*), we again find agreement between the experimental and fitted scattering curves (χ^2^ = 0.08). The corresponding real-space reconstruction (Fig. 5 ▸ *b*) presents two independent tapes within the building block (see Fig. S17*a* for point representations). However, the two tapes do not appear to be symmetric along the *z* direction, suggesting a displacement angle of φ 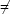 180° between them. The cross section of the helical tapes presents a rather rough surface that does not allow more detailed interpretation, as is typical for such random-movement DA models. Nevertheless, a comparison of the reconstruction with the model according to the previously published dimensions (Kornmueller *et al.*, 2015 ▸) gives good agreement (see the red model in Fig. 5 ▸ *c*). A movie of the rotating helices can be found in the supporting information. To obtain a measure of the uniqueness of the final model, we repeated the fitting procedure to end up with 16 independent reconstructions. The average of all 16 models (Volkov & Svergun, 2003 ▸), as shown in Fig. S18(*a*), is consistent with the single reconstruction. In agreement with the reference values shown in Fig. 3 ▸, the 〈NSD〉 was found to be 1.27 ± 0.02, hence confirming the reproducibility of the reconstruction.

Analogous to the above, we further applied the reconstruction procedure to experimental data for the globular protein alcohol de­hydrogenase 1 (ADH) in phosphate-buffered saline at pH 7.5. The data set was taken from the SASBDB database (Valentini *et al.*, 2015 ▸; date of download 12 December 2016), corresponding to the identifier SASDA52. Again, we first determined the size of the initial random configuration (*d* = 9 nm) from the PDDF, as seen on the right of Fig. 5 ▸. We then fitted the scattering data over the angular range 0.13 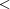 *q* 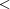 6 nm^−1^, corresponding to a real-space resolution of approximately 0.5 nm. The default fitting parameters (*M* = 1) according to Section 4 were used, but this time the number of DAs was increased to 1500, due to the outstanding angular range.

Also in this case, the experimental data were fully fitted throughout the reconstruction procedure (χ^2^ = 0.24; see Fig. 5 ▸ *c*). Interestingly, the corresponding real-space model (Fig. 5 ▸ *d*) presents a characteristic triangular cross section that is recurrent from different perspectives (see Fig. S17*b* for point representations). We thus compared the reconstruction with the ADH crystal structure found in the literature (Raj *et al.*, 2014 ▸), and an overlay of the two models can be seen in Fig. 5 ▸(*d*). Undoubtedly, the reconstructed model is qualitatively in agreement with the molecular structure; quantitatively, we find an NSD between the crystal structure and the reconstruction of 0.93. Also in this case, we performed a total of 16 independent reconstructions (〈NSD〉 of 1.20 ± 0.02), where the averaged representation is consistent with the molecular structure (see Fig. S18*b*).

This, in congruence with the previous example, demonstrates the applicability of the proposed fitting algorithm for the real-space reconstruction of seemingly infinite and globular geometries from experimental small-angle scattering data.

## 7. Conclusions   

In conclusion, we have presented a new method for the reconstruction of the structural motif of seemingly endless rod-like systems, such as helices. In this regard, the following points were critical:

(i) We optimized the numerical complexity of the Debye formula for the unique case of recurrent symmetry along the elongation direction, resulting in the proposed projection scheme.

(ii) Based on this projection scheme, we developed a metaheuristic fitting algorithm. Instead of minimizing a variety of structural penalties (Franke & Svergun, 2009 ▸), the system is repeatedly forced out of the current numerical equilibrium by forcing the DA to move, regardless of the damage caused.

(iii) The algorithm is implemented in the multi-platform compatible graphical computer program *SasHel*, which allows live tracking of the fitting progress in real and reciprocal space and encourages user interaction.

We have demonstrated the functionality and reliability of the presented method using a variety of analytical and experimental examples. These showcases provide a comprehensive reference for future users. We have further addressed and discussed the risks of wrongly chosen fitting parameters or insufficient data quality: we have illustrated a series of negative examples to discuss which reconstructed features might be true or not (see Section S2 and Figs. S7–S11 and S19–S26). *SasHel*, the computer program corresponding to this work, also includes an option for shape reconstruction of globular particles. This, in congruence with its originally intended use, makes the program applicable over a wide range of SAXS-based structural studies, expanding the scope of currently available dummy-atom modelling software.

The program *SasHel* is freely available for academic use. The most up-to-date version can be obtained from http://sashel.tugraz.at or upon request from the authors.

## 8. Related literature   

The following references are cited in the supporting information: Damaschun *et al.* (1968 ▸); Konarev & Svergun (2015 ▸); Shannon & Weaver (1949 ▸); Taupin & Luzzati (1982 ▸).

## Supplementary Material

Additional discussion and figures. DOI: 10.1107/S2052252518005493/tj5016sup1.pdf

Supporting movie: rotation of the reconstruction from experimental data as shown in Fig. 5(b). DOI: 10.1107/S2052252518005493/tj5016sup2.avi

## Figures and Tables

**Table 1:** Main concepts of the proposed grid-free fitting algorithm, the corresponding mathematical parameters, their function and their implementation compared with current solutions in fixed-grid DA modelling programs For details of current fixed-grid methods, see Svergun (1999 ▸), Franke & Svergun (2009 ▸) and Koutsioubas & Pérez (2013 ▸).

Concept	Fixed-grid methods	Parameter	Function	Implementation
Scalability	Grid size	*D_X_*	Scaling parameter	*d*_N12_, RC(*X*), random movement generator
Homogeneity	Fixed grid/looseness penalty	*d*_N12_	Avoid DA clustering/disconnected DAs	Movement classifier/forced reconfiguration
Compactness	Looseness penalty/limited search volume	RC(*X*)/*D*_*X*,crit_	Keep DAs close to (radial) centre of mass/avoid model explosion	Regularization term/forced reconfiguration

**Table 2:** Detailed dimensions of the models shown in Figs. 3 ▸ and 4 ▸; explanations of the model variables can be found in the corresponding references

	Model											
Parameter	*A*	*B*	*C*	*D*	*E*	*F*	*G*	*H*	*I*	*J*	*K*	*L*
Reference	(*a*)	(*a*)	(*a*)	(*b*)	(*b*)	(*b*)	(*b*)	(*a*)	(*a*)	(*a*)	(*c*)	(*d*)
*R*_o_ (nm)	10	10		10	10	10	10	10	10		10	10
*R*_i_ (nm)	0	5		5	0	5	0		5			5
*a*, *b* (nm)		.	8, 20							15		
*L* = *c* (nm)	1000	1000	1000	1000	1000	1000	1000			15	5	
*P* (nm)				50	50	50	50					
ω (°)				45	6	45	6					
φ (°)						180	180					

References: (*a*) Feigin & Svergun (1987 ▸), (*b*) Pringle & Schmidt (1971 ▸), (*c*) Guinier & Fournet (1955 ▸), (*d*) Kawaguchi (2001 ▸).

## Appendix A Model calculations

All scattering patterns shown throughout this work were calculated according to the literature, with the exact dimensions and references shown in Table 2 ▸. The error band of each data set was estimated according to σ(*q*) = 0.2(*cm* ^−1/2^)[*I*(*q*)]^1/2^ (see Section S2 and Figs. S19–S23 for test cases with noisy data). For models *A*–*L* we used a total of 124 data points in the angular range 0.01  *q*  2 nm^−1^. In case of seemingly endless geometries, we further ensured that the stacking distance *H*_BB_ was resolvable in the lower angular limit (*H*_BB_  π/*q*_min_). All PDDFs throughout this work were calculated using the *GIFT* software package (Bergmann *et al.*, 2000 ▸).

All reconstructions were performed on a ‘standard work station’ from Hewlett–Packard, using an Intel Core i7 4800MQ processor with four cores, each operating at 2.7 GHz.
