# Supplementary material for: Dummy-atom modelling of stacked and helical nanostructures from solution scattering data
Source: IUCrJ. 2018 May 11;5(Pt 4):390–401. doi: 10.1107/S2052252518005493 (PMC6038956; doi:10.1107/S2052252518005493)
Supplement: Supplementary file 1 [file m-05-00390-sup1.pdf]

IUCrJ

Volume 5 (2018)

Supporting information for article:

Dummy-atom modelling of stacked and helical nanostructures from solution scattering data

Max Burian and Heinz Amenitsch

S1. Determination of the stacking distance – details and limitations

S1.1. Fitting the dPDDF

One of the key structural parameters of stacked or helical structures is the so-called repeat distance or helical pitch, respectively, which, in the context of this work, is the stacking-distance between building-blocks along the elongation direction in the proposed projection scheme (see main Figure 1). This recurrent nature of the same motif causes oscillations in the high-r regime of the pair distance distribution function (PDDF), as e.g. shown in Supporting Figures S1-3. However, these oscillation peaks are damped due to a sloped decay because of convolution with the overall shape. In the ideal case of perfectly 1D elongated structures such as stacked spheres the decay is linear (see Supporting Figure S1). We hence use the numerical derivative of the PDDF (dPDDF), to filter-out this decays its derivative yields constant offset for 1D elongated structures. However, additional distortions and damping-effects of the high-r oscillations can occur, such as bending of the structure, non-linearity of the helical strands, 1st and 2nd type of disorder in the stacking distance, etc. To account for we therefore fit the dPDDF using the damped sinusoidal function

$$f(r) = y_0 + A \cdot \sin\left(\frac{2\pi}{H_{BB}} \cdot r + \varphi_0\right) e^{-\tau r} \qquad (S1)$$

Here, the frequency is directly related to the stacking-distance of the building blocks H_{BB}, whereas the other parameters y_0, A, φ_0 and τ are equally determined but yield no information relevant for this work.

S1.2. The PDDF Fourier limit

In general terms, the pair distance distribution function (PDDF) is the Fourier transform of scattering data, converting the information content from reciprocal into real-space (Glatter & Kratky, 1982; Feigin & Svergun, 1987). For an accurate calculation, this Fourier transform needs to be calculated over the full angular regime $0 < q < \infty$. However, the angular range in a given experimental dataset is limited by the smallest and largest accessible scattering vector q_{min} and q_{max}, which act as Fourier limits. As a consequence, the e.g. largest resolvable real-space dimension in the PDDF is limited by $D_{max} < \pi/q_{min}$. The numerical calculation of the PDDF thus requires a boundary condition such that $PDDF(r) = 0$ for $r > D_{max}$ (Glatter & Kratky, 1982; Feigin & Svergun, 1987). Yet, in the case of seemingly infinite systems as discussed in this work, the length of a given structure L is larger than the resolvable limit $L > \pi/q_{min}$ in reciprocal space or $L > D_{max}$ in real-space, which violates the boundary condition above.

In order to investigate if the PDDF can be obtained from scattering data even if $L > D_{max}$, we calculated the scattering pattern of 63 concentric stacked rings (see model $\boldsymbol{L}$ in Table 2) using a d-spacing of $7nm$ between them, which results in a total height of 439 nm. We then obtained the PDDF from this data-set using an increasing low-q Fourier limit q_{min} such that simultaneously, the resolvable limit D_{max} is decreased. The resulting curves, as well as the dPDDFs with corresponding fits are shown in Supporting Figure S3.

Of course, the low-q transition from the q^{-0} Guinier to the rod-like q^{-1} Porod regime in the PDDF fits of the scattering patterns occurs earlier the more the scattering curve is truncated (see Supporting Figure S2 - top). However, the resulting PDDFs and dPDDFs remain congruent as only the number of stacking induced oscillations decreases due to the artificial truncation of the low-q range and consequently $D_{max} = \pi/q_{min}$. Further, and most important for the scope of this work, the corresponding fits of the dPDDF yield the right stacking-distance with an approximate error of <2%. A model reconstruction from the used scattering pattern can be found in Supporting Figure S5.

S1.3. Limitations and error of misdetermination

Of course, the determination of the stacking-distance from the dPDDF relies on the fact that the stacking-induced oscillations can be separated from the single-building-block- and the cross-sectional-fingerprint. In the case of helical structures, there exist two scenarios where this might not be the case – for extremely high (case one) and extremely low (case two) building-block aspect ratios. Moreover, in case three, the effect of a wrongfully determined stacking-distance H_{BB} on the reconstruction is discussed.

For case one, if the helix is *pulled apart,* the stacking-distance becomes significantly larger than the helix diameter: the actual twisted motif becomes weaker and less influential until, eventually, the helix is completely straight. Hence, the cross-sectional-fingerprint dominates the PDDF while the stacking-induced oscillations disappear. To investigate the possible error resulting from this effect, we determined the stacking-distance from the dPDDF of helices according to model $\boldsymbol{D}$ in Figures 2 and 3 with increasing pitch, ranging from 10 to 100 nm (in all cases with diameter of 20 nm). The corresponding scattering curves, PDDFs, dPDDFs and fits can be found in Supporting Figure S5. For all cases with a stacking distance $H_{BB} < 70$ nm, the error of misdetermination was less than 2%, while this error systematically rises up to 6% for larger aspect ratios.

The other scenario (case two) in which the stacking-induced oscillations might be influenced by the building-block is the opposite case of a highly compressed helix. In these cases, the stacking-distance is smaller than the helix-diameter, hence convoluting the first 1-2 high-r oscillations. This can cause misinterpretation, if these peaks are not neglected when fitting the damped sine function to retrieve the stacking-distance. As an example we fitted the dPDDF of the helix in model $\boldsymbol{D}$ in Figures 2 and 3 with a diameter of 20 nm and a pitch of 10 nm. As seen in Supporting Figure S6, the determined

stacking-distance changes from 9.83 to 9.64 nm, if the first peak is considered, thus leading to an increased error.

However, case three, there always exists the possibility of choosing a (slightly-) wrong building-block stacking-distance, e.g. when noisy experimental data is used (see Supporting Information section S2 for details). Hence, the question arises to which extend the reconstruction remains stable. We thus took the scattering patterns of models $\boldsymbol{D}$ and $\boldsymbol{F}$ (pitch of 50 nm) and reconstructed the building-block motif using a series of wrong stacking-distances, ranging from $30 - 70$ nm. Each reconstruction was performed under the exact same conditions in order to ensure comparability. The fitted scattering curves as well as the final reconstructions are shown in Supporting Figures S7-10.

In case of model $\boldsymbol{D}$: On first sight, the fitted scattering patterns are in good agreement with the analytical model. Yet, a detailed look at the low angular regime reveals clear deviations for the used stacking-distances $30, 40, 60$ and $70nm$ (see Supporting Figure S7). Similarly, the corresponding reconstructions present significant artefacts both in the lateral and cross-sectional perspective. As one would expect, these deviations in real- and reciprocal-space become smaller the closer the used stacking-distance comes to the real dimension (50 nm). Even though both the fitted scattering curves and the reconstructions of the cases using 45 and 55 nm still show deviations from the ideal case, the motif of the structure being a continuous single-strand helix, remains preserved.

In case of model $\boldsymbol{F}$: In this case, the fitted scattering patterns are in good agreement with the analytical model over the full q-range. Even in the low-q range, no particular deviations can be found (see Supporting Figure S9). Interestingly, the reconstructions in all cases present the typical double strand motif, even in cases of 30 and 70 nm (model pitch of 50 nm). However, particularly these two cases present strong deviations from the ideal shape in regard of the continuity of the helical strands. It is further noteworthy, that in none of the reconstructions of model $\boldsymbol{F}$ one helical strand is higher populated than the other, as one would expect if a helical motif would be forced onto the system. The helical bias term ($\gamma = 0.3$ for all reconstructions shown in this work) hence does not influence the final reconstruction.

These illustrative examples suggest that the algorithm presented in this work supplies stable reconstructions even if the stacking-distance of the building-blocks is determined with a relative error of less than 10%.

S2. Additional considerations on “model resolution”

The terminology of “model resolution” used in this work relates to two major aspects: the resolution limited by the information content provided by the scattering curve as well as the resolution relating to the used number of dummy atoms (DA). While these aspects overlap to some extent, we briefly address them separately to raise awareness and prevent model over- and misinterpretation.

S2.1. Angular information content

A quantitative estimate of the information content accessible from scattering data is the number of Shannon channels N_S. In the case of globular particles, this number is defined by the largest accessible scattering angle q_{max} and the maximum real-space dimension D_{max} by $N_S = q_{max}D_{max}/\pi$ (Shannon & Weaver, 1949; Damaschun *et al.*, 1968; Taupin & Luzatti, 1982). Nevertheless, for helical structures, D_{max} is seemingly infinite, which makes this formalism impractical.

In a similar but more general formalism, q_{max} can also be used to estimate the minimal sampling-distance in real-space. This sampling distance hence defines the, mathematically speaking, real-space resolution corresponding to the scattering curve, such that the smallest resolvable feature is $d_{min} > \pi/q_{max}$ (Glatter & Kratky, 1982; Feigin & Svergun, 1987). In the case of scattering from a helical structure, the smallest structural motif is the width of the helical tape cross-section. Thus, the question arises to which extent the reconstruction of this motif is affected by an insufficient angular range and therefore real-space resolution.

To visually investigate this effect, we repeated the reconstructions of models ***D*** and ***F*** in Figures 2 and 3, using scattering curves with a de- and increased angular range. The resulting models and the corresponding curves are shown in Supporting Figure S19 & 20. In case of model ***D***, all three reconstructions present the characteristic helical motif. However, the quality of the reconstructed tape as well as the overall radial cross-section is evidently better with increasing q-range. Also in case of model ***F***, all three reconstructions present the characteristic double strand motif, whereas again the quality of the reconstructed strands as well as the overall radial cross-section is better with increasing q-range. Interestingly, even in the low resolution case (***S.Fa***) both helical strands are equally populated, suggesting that the helical bias parameter γ does not influence the reconstruction.

In relative terms, the helical-tape cross section of the used models is $t_w = 5$ nm whereas the resolution defined by the scattering angle corresponds to 3.1, 1.6 and 1.0 nm in the case of model scattering ***S.Da/S.Fa***, ***S.Db/S.Fb*** and ***S.Dc/S.Fc.***, respectively. As only the latter two reconstructions (***S.Db/S.Fb*** and ***S.Dc/S.Fc***) resemble the actual model shape, we can conclude that reconstruction is not feasible if the resolution defined by the scattering range is worse than half of the helical-tape cross section.

S2.2. Statistical information content

Experimental scattering data is never noise free. Hence, the information content provided by a scattering curve is not only limited by the experimentally accessible angular range but also by the statistical quality of the data: the noise (Konarev & Svergun, 2015). So the obvious question in regard to this work is, at which noise level the determination of the stacking-distance and the fitting algorithm break down?

Therefore, we took the reference patterns of models ***D*** and ***F*** with an angular range corresponding to case ***S.Db/S.Fb*** ($q_{max} = 2\ \mathrm{nm}^{-1}$) and gradually added artificial noise. In detail, we defined the error-band according to $\sigma(q) = E\ [cm^{-\frac{1}{2}}] \cdot \sqrt{I(q)}$, whereas the four model cases ***S.D5/S.F5, S.D10/S.F10, S.D50/S.F50*** and ***S.D100/S.F100*** correspond to E=5, 10, 50 and 100, respectively. We then distorted the scattering patterns by randomly moving each data point according to a Gaussian distribution with a standard-deviation defined by the error band. At $q_{max} = 2\ \mathrm{nm}^{-1}$, the relative noise compared to the scattering signal is hence approx. 15, 30, 150 and 300 % for ***S.D5/S.F5, S.D10/S.F10, S.D50/S.F50*** and ***S.D100/S.F100***, respectively. The scattering curves together with the PDDFs and dPDDFs can be found in Supporting Figure S21.

We first used the noisy data sets to determine the stacking distance H_{BB}. For cases ***S.D5, S.D10*** and ***S.D50*** the found building-block height is within 10% of the real value (see Supporting Figure S21). Only in the case of ***S.D100***, we find a stacking-distance of 56.89 nm, which is off by 14 % compared to the model-pitch of 50 nm. For cases ***S.F5-100***, the building-block height is within 10% of the real value (see Supporting Figure S16). However, in case of ***S.F100***, only two oscillations are found in the dPDDF, yielding the determined value questionable.

Nevertheless, we reconstruct the structural motif from all scattering patterns using the determined stacking-distances. As seen in Supporting Figure S22, starting from the case of ***S.D10*** the helical strand is not anymore continuous in the reconstructed model – a phenomenon similar to the effect of insufficient angular resolution as observed for ***S.Da*** in Supporting Figure S19. However, as seen in the point-representations in Supporting Figure S22c, the DA density remains homogenous such that a helical motif is suggested. In the cases of ***S.D50*** and ***S.D100*** excessive DA clustering is observable such that the reconstruction is not feasible. However, a detailed look at the model data and the fitted scattering patterns in Supporting Figure S22a of ***S.D10, S.D50*** and ***S.D100*** shows an interesting anomaly: the reconstruction algorithm wrongfully fits artefacts in the scattering patterns that are caused by the random noise. In order to avoid this circumstance, we used the scattering patterns of the fitted PDDF curves (see Supporting Figure S21) as input for the reconstruction procedure. As shown in point representations in Supporting Figure S23 all reconstructions now present a homogenous DA distribution and no clustering occurs. However, a continuous helical strand is only found for cases ***S.D5, S.D10*** and ***S.D50.***

Similar phenomena are witnessed for model ***F***: as shown in Supporting Figure S24, direct fitting of the scattering data yields the characteristic double-stand motif for all reconstructions. For ***S.F50*** and ***S.F100,*** minor deviations of the single strands from the ideal shape can be observed. In contrary to model ***D***, reconstructions from the scattering patterns of the fitted PDF curves (see Supporting Figure S16) show no significant improvement or worsening (see Supporting Figure S25). However, a detailed comparison of the point plots (Supporting Figures S24c & 25c) reveals a more homogenous

DA distribution within the helical strands in the latter case (using the scattering intensity of the fitted PDDF). We find it further noteworthy that also in this case, no influence of the helical field can be seen in any of the double strand helix reconstructions.

To summarize the above findings, direct fitting of noisy experimental data can lead to physically unfeasible artefacts such as DA clustering or strong inhomogeneties in the DA density, which can be improved using the smoothed scattering curve from the PDDF determination.

S2.3. DA resolution

In the case of fixed-grid DA modelling (Chacón *et al.*, 1998; Walther *et al.*, 2000; Svergun, 1999; Franke & Svergun, 2009; Koutsioubas & Pérez, 2013), the scale of the grid and thus the model resolution is defined *prior* to the fitting process. Hence, the number of DAs that represent the final model solely depends on the shape of the reconstruction. However, in the case of fitting by random DA movement, as proposed in this work, such an *intrinsic* spatial resolution limit does not exist. Consequently, to increase the model resolution, it appears intuitive to simply increase the number of DAs used for the fitting process, as this would add additional degrees of freedom to e.g. model the structure's surface. However, and as already written in section 4.4, when performing such a random movement model reconstruction, it can be expected to end up with a fitted configuration that presents structural features below the resolution limit of the experimental data ($d_{min} = \pi/q_{max}$ where q_{max} is the upper angular range of the fitable data (Glatter & Kratky, 1982; Feigin & Svergun, 1987)). Excessively increasing the number of DAs might thus actually not necessarily increase the model resolution, as also the amount of non-resolvable model artefacts will increase. To minimize the risk of over-interpreting such artefacts, we suggest to keep the number of DAs rather low (from experience $N < 1000$) or, if large numbers cannot be avoided, to always compare the final model with the resolution limit provided by the scattering curve and to perform a statistical analysis using e.g. DAMAVER (Volkov & Svergun, 2003) (see Supporting Figure S18 for an example of such an analysis).

However, when minimizing the number of DAs used for the fitting process, one might run into other issues such as general under-sampling or, in the case of the proposed *projection scheme*, coherence effects resulting from the mirroring of an exact DA conformation. To illustrate the latter effect, we calculated scattering curves of artificially constructed single-strand helices with a decreasing number of DAs. In one case we generated the helix (consisting of 15 building block units) by randomly filling up the *full* helix, such that no two building-blocks would be the same. We then used the standard Debye formula to obtain the scattering pattern. In the other case, we only filled up one single building-block and then used our *projection scheme* to calculate the scattering curves. The results as well as the corresponding analytical model (Pringle & Schmidt, 1970) are shown in Supporting Figure S26.

As expected, the lower angular regime of all model curves ($q < 1\ \mathrm{nm}^{-1}$) is in good agreement with the analytical one. Even in the case of the $250\ \mathrm{DAs/BB}$ model, which corresponds to an approximate next-neighbor DA distance of 3 nm, no significant deviations are visible. Yet, when looking at the upper angular regime ($1 < q < 2\ \mathrm{nm}^{-1}$) of the *projection scheme* curve ($250\ D\mathrm{As/BB}$), two rather obvious peaks appear. If now the number of DAs is increased, these peaks become weaker ($500\ \mathrm{DAs/BB}$) and eventually disappear($1000\ D\mathrm{As/BB}$). However, the sole fact that the peaks' positions between 250 and the $500\ \mathrm{DAs/BB}$ model do not change indicates that this effect is caused by the stacking of identical building blocks. A comparison to the scattering curves from the fully filled helices (equivalent to the stacking of non-identical building blocks) confirms this, as no such peaks can be found in the $250\ D\mathrm{As/BB}$ model.

To summarize the above, an excessive increase of the number of DAs used in a fitting process causes not only an immense numerical overhead, but also leads to an increased number of non-resolvable artefacts. This can further lead to misinterpretation of reconstructed models. On the other hand, if coherence effects, such as depicted in Supporting Figure S26, are visible in the fitted curve, one should nevertheless consider increasing the number DAs to suppress these effects.

S3. Fitting algorithm – technical details

The fitting algorithm implemented in SasHel starts from a random configuration X_0. If wanted (but not suggested) the user can alter the following parameters (default values given in brackets): the initial temperature T_0 (1), quenching coefficient q_T (0.99), number of iterations $N_k(100)$, number of fails $N_{fails}(100)$, helical bias parameter $\gamma(0.3)$ and radial compactness weight $\beta(1)$. The functional $f(X)$ and the scaling paramter D_X of a given configuration X are evaluated according to equations 8 and 4, respectively. Random movements $\boldsymbol{r_{rand}}(T)$ are generated according to equations 5 and 6. Each random movement is checked 100 times for a collision via the hard contact limit $0.1\ \langle d_{\mathrm{N12,X}}\rangle$. To recall:

- $\langle d_{\mathrm{N12,X}}\rangle$ denotes the average d_{N12} parameter over configuration X,
- $|\boldsymbol{r_i}|$ denotes the distance of the ith DA to the center of mass (COM) (in case of stacked structures only $\boldsymbol{e_x}$ and $\boldsymbol{e_y}$ directions are considered),
- $\langle|\boldsymbol{r}|\rangle_X$ denotes the *mean distance of all DAs* to the COM (in case of stacked structures only $\boldsymbol{e_x}$ and $\boldsymbol{e_y}$ directions are considered) and
- $\boldsymbol{r_{NN,i}}(x)$ denotes the vector from the ith DA to the COM of the x nearest neighbors of i. As an example, $|\boldsymbol{r_{NN,i}}(1)|$ returns the distance of the ith DA to the nearest neighbor.

A pseudo-code implementation of the full fitting algorithm is shown in Supporting Figure S27.

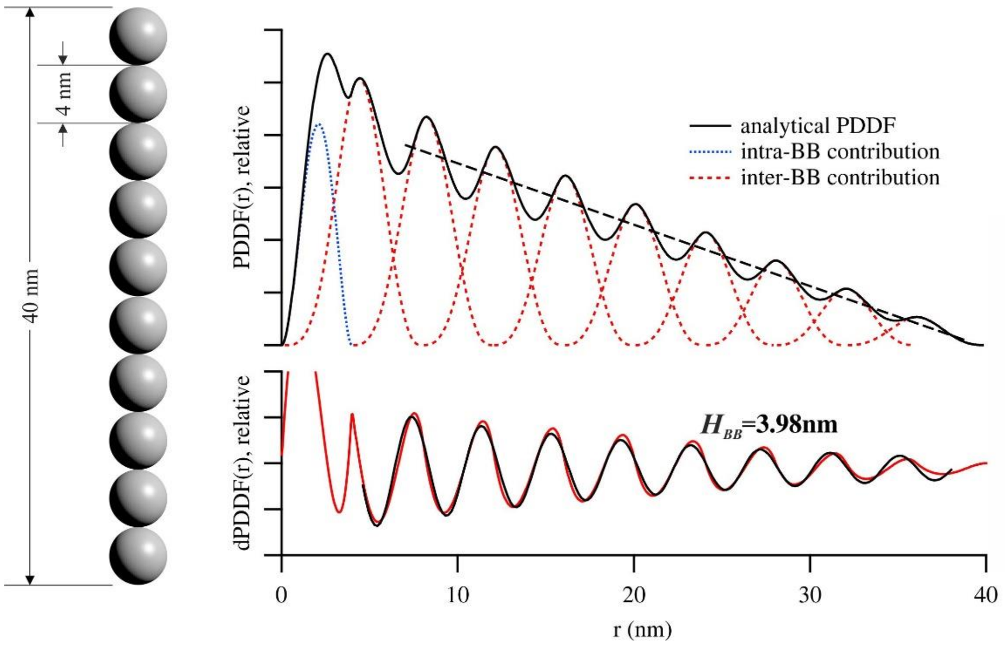

Figure S1 Analytical investigation of phenomena occurring in the PDDF and dPDDF using a model-case of 10 spheres with diameter of 4 nm and a stacking distance of 4 nm (Glatter, 1980). An illustration of the model used as input for the calculation of the PDDF is shown on the left. The calculated PDDF on the right shows distinct peaks, correlating to the intra- and inter-building-block contributions (blue and red, respectively). The dashed black line shows the linear nature of the high-r decay, characteristic for 1D extended geometries (Feigin & Svergun, 1987). The damped sine-function fit of the numerically calculated dPDDF on the bottom is in good agreement ($H_{BB} = 3.98$ nm compared to input stacking distance of 4 nm).

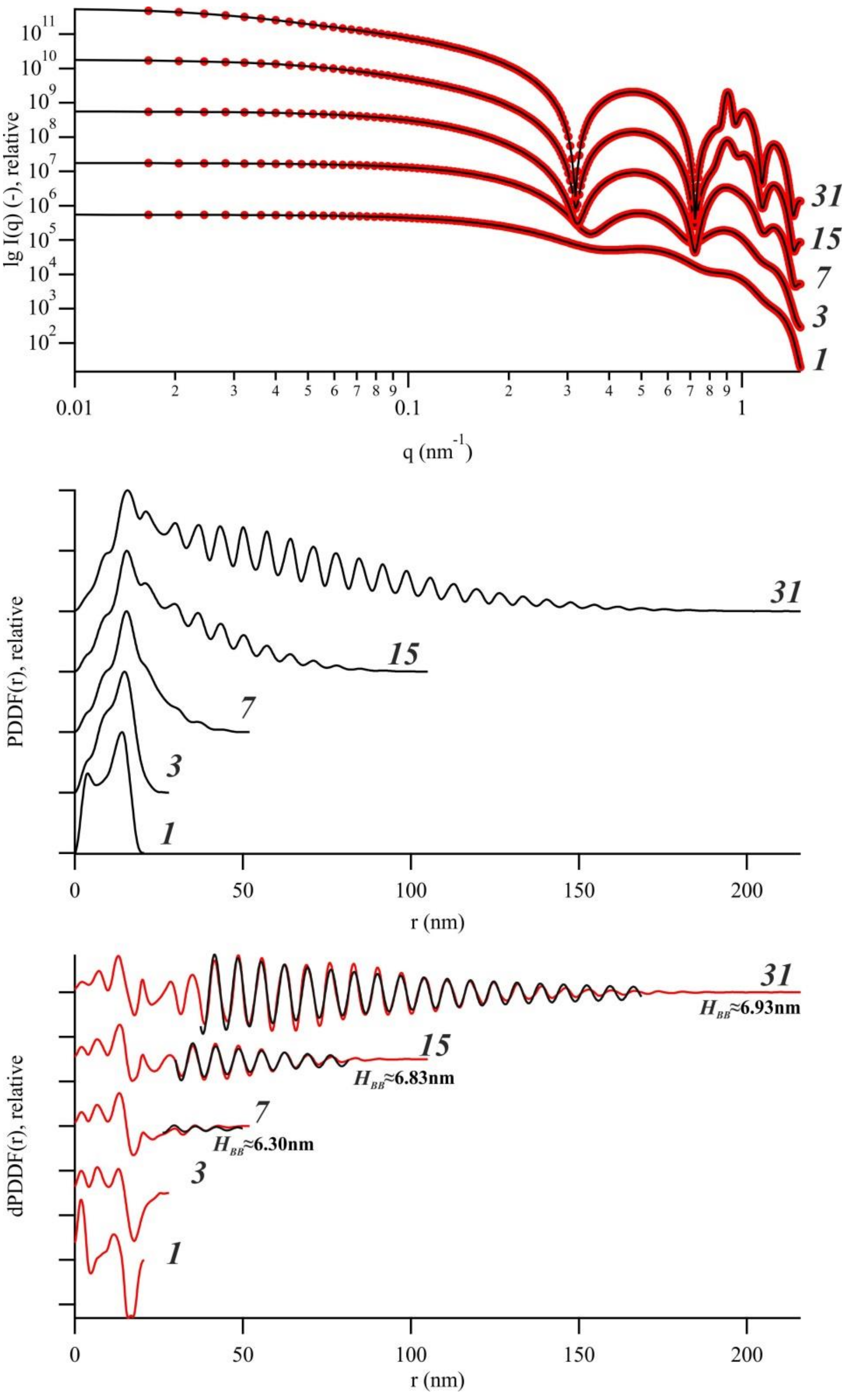

Figure S2 Evolution of stacking-induced oscillations in the PDDF for the case of stacked torus. (Top) Scattering curves of stacked torus models (see model ***L*** in Table 2 of the main text) with a d-spacing of 7 nm (red dots) together with the PDDF fits (black lines). The specified number denotes the number of rings that are axially stacked with a distance of 7 nm of each-other. (Middle): PDDFs of the corresponding scattering curves. (Bottom) Derivatives of the PDDFs (dPDDFs – red lines) as well as the fitted sinusoidal functions from which the stacking distances are determined (black lines).

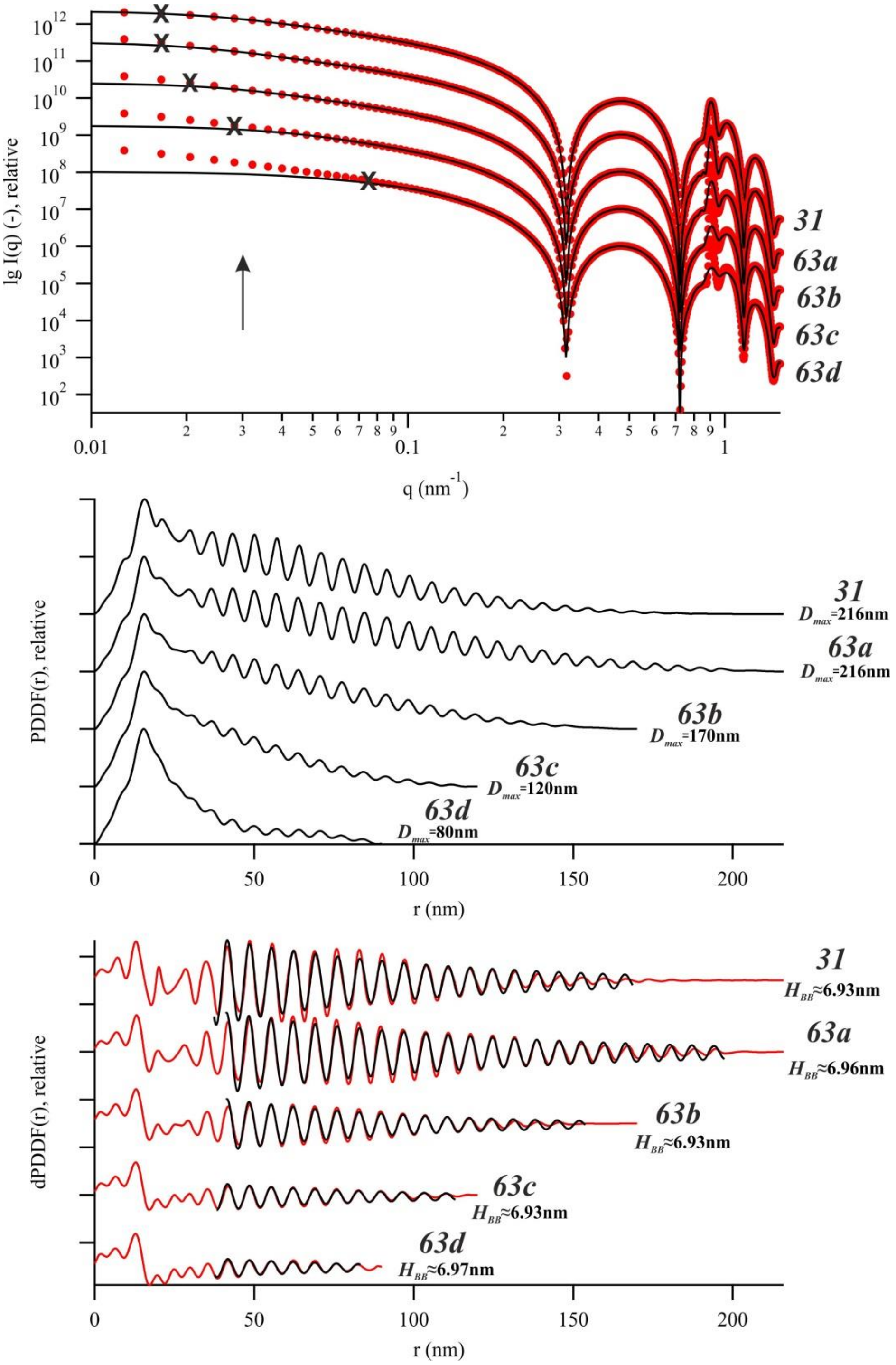

Figure S3 Effect of a truncating Fourier Limit. (Top) Scattering curves of stacked rings (see model ***L*** in Table 2 of the main text) with a d-spacing of 7 nm (red dots) together with the PDDF fits (black lines). Transition from Guinier to Porod regime is indicated by an arrow. The specified number denotes the number of rings that are stacked on each-other. The black crosses indicate the q_{min} used for the calculation of the PDDFs (Middle): PDDFs of the corresponding scattering curves using the D_{max} as specified. (Bottom) Derivatives of the PDDFs (dPDDFs – red lines) as well as the fitted sinusoidal functions from which the stacking distances are determined (black lines).

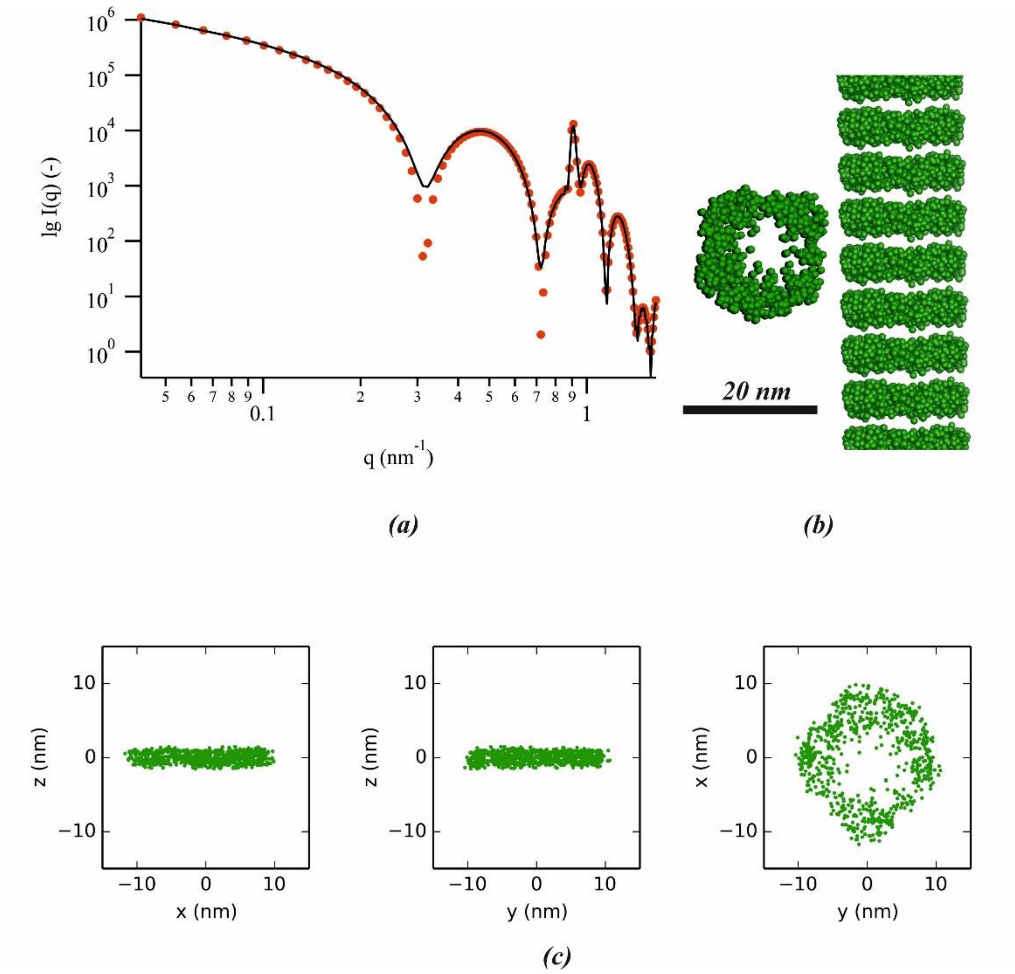

Figure S4 Reconstruction from the scattering curve (curve ***63b***) of the stacked-rings model used in Supporting Figures S1 and S2. The rings correspond to model ***L*** in Table 2 of the main text and are separated by a d spacing of 7 nm. In this case, the reconstruction was performed from the scattering curve corresponding to 63 stacks (red dots) whereas the fitted curve (black line) is in good agreement with the theoretical model data. The reconstruction clearly resembles the structural motif of the model, further validating the algorithm proposed in this work.

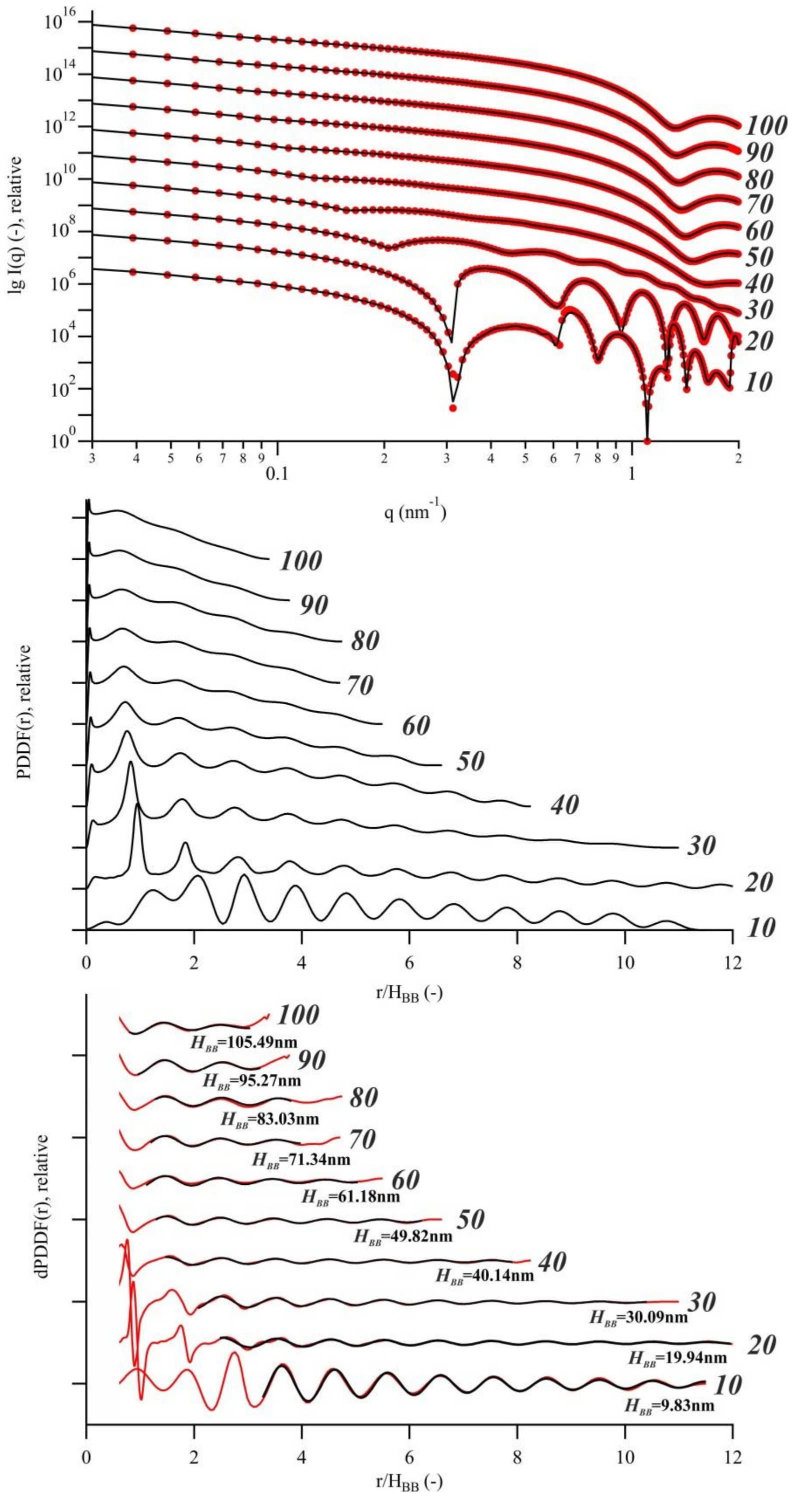

Figure S5 Influence of increasing helical pitch on the determined stacking distance. (Top) Scattering curves of single strand helices (see model ***D*** in Table 2 of the main text) with varying pitch (red dots) together with the PDDF fits (black lines). The specified number denotes the helical pitch, ranging from 10 to 100 nm. (Middle): PDDFs of the corresponding scattering curves. (Bottom) Derivatives of the PDDFs (dPDDFs – red lines) as well as the fitted sinusoidal functions from which the stacking distances are determined (black lines).

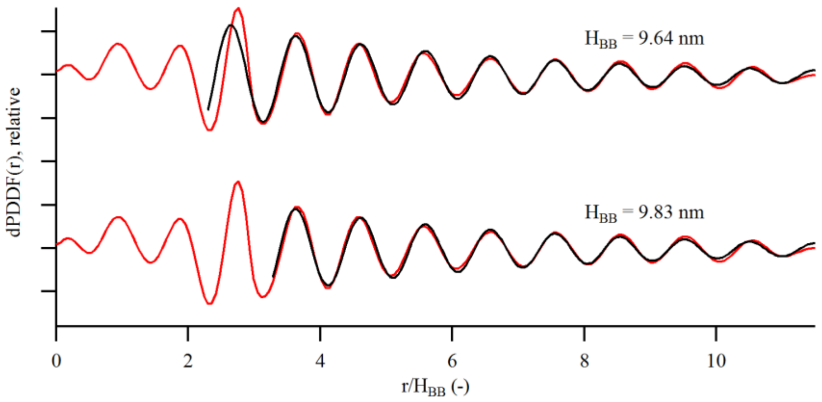

Figure S6 Derivative of the PDDF (dPDDF) of a single strand helix as model ***D*** with a pitch of $10\ nm$ (red lines) as well as two independently fitted sinusoidal functions from which the stacking distance can be determined (black line). At such a low pitch-to-diameter ratio, the first stacking-induced oscillations can be influenced by the building-block characteristic PDDF-fingerprint. This can cause a distortion on the fitting procedure. Inclusion of this peak thus leads to an increased error (top) in the determined stacking-distance. The scattering curve as well as the PDDF corresponding to the dPDDF can be found in Supporting Figure S4.

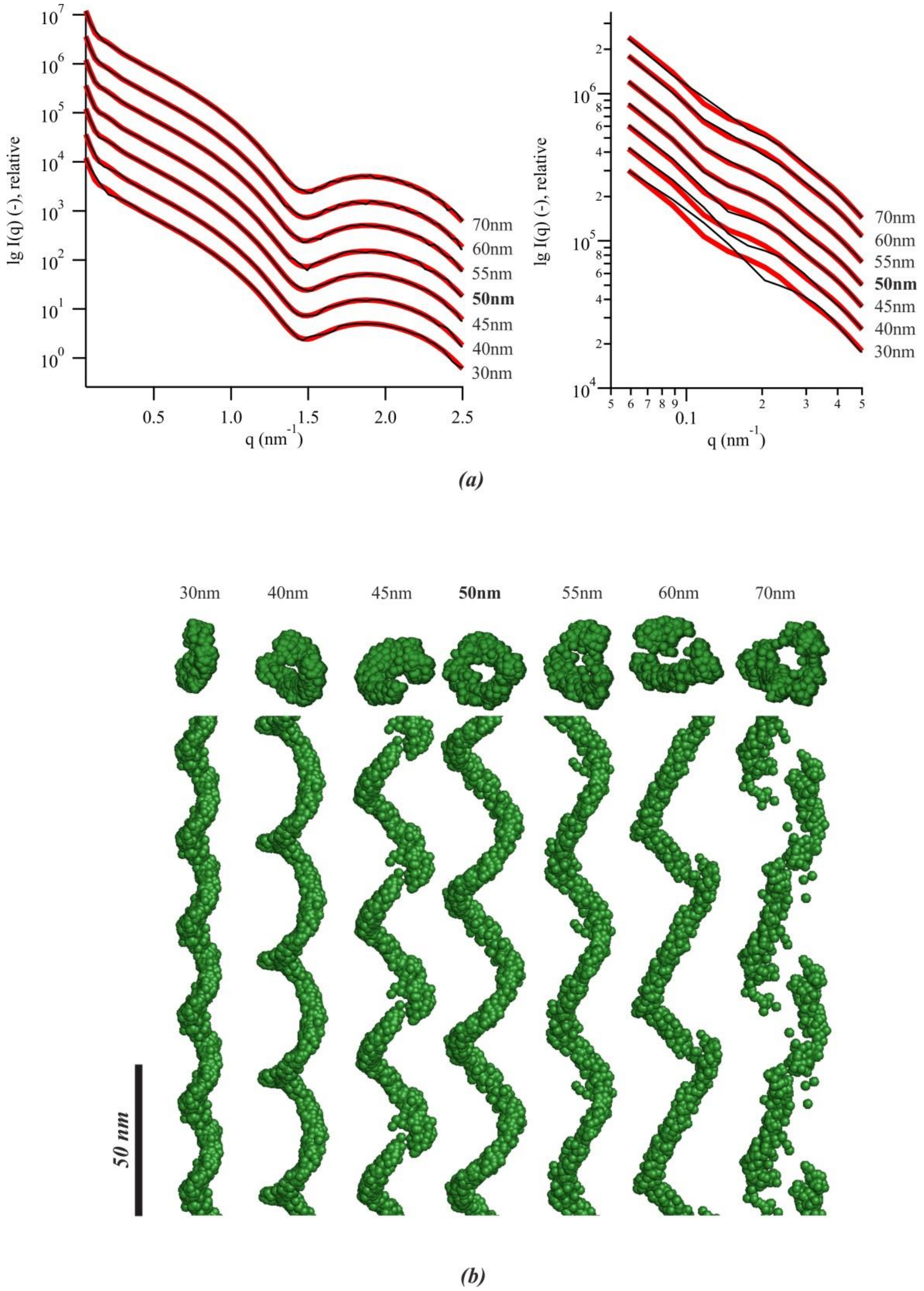

Figure S7 Reconstruction of model ***D*** (see Table 2 of the main text) with a building-block height of $50nm$ using a series of wrong building-block stack distances H_{BB}. The fitted curves are shown in (a): while all fits appear acceptable on first sight, larger deviations can be found in the low-q regime (see inset). Further, the actual reconstructions shown in (b) present significant artefacts if H_{BB} deviates by more than 10% from the real value. Point-representations of the reconstructions can be found in Supporting Figure S8.

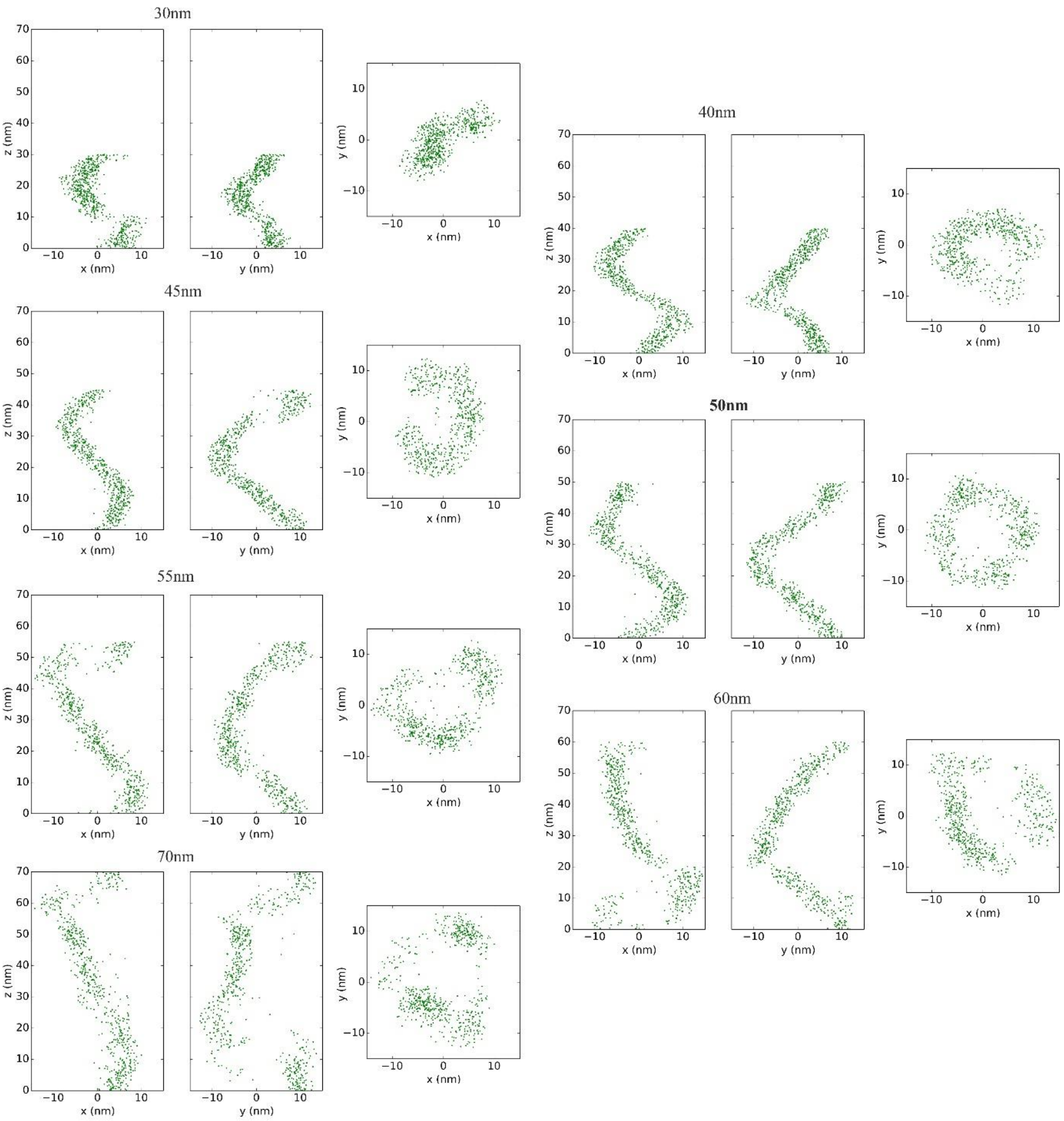

Figure S8 Point-representations corresponding to Supporting Figure S7, showing orthogonal views of the reconstructions of model ***D*** using a wrongly chosen stacking distance H_{BB}.

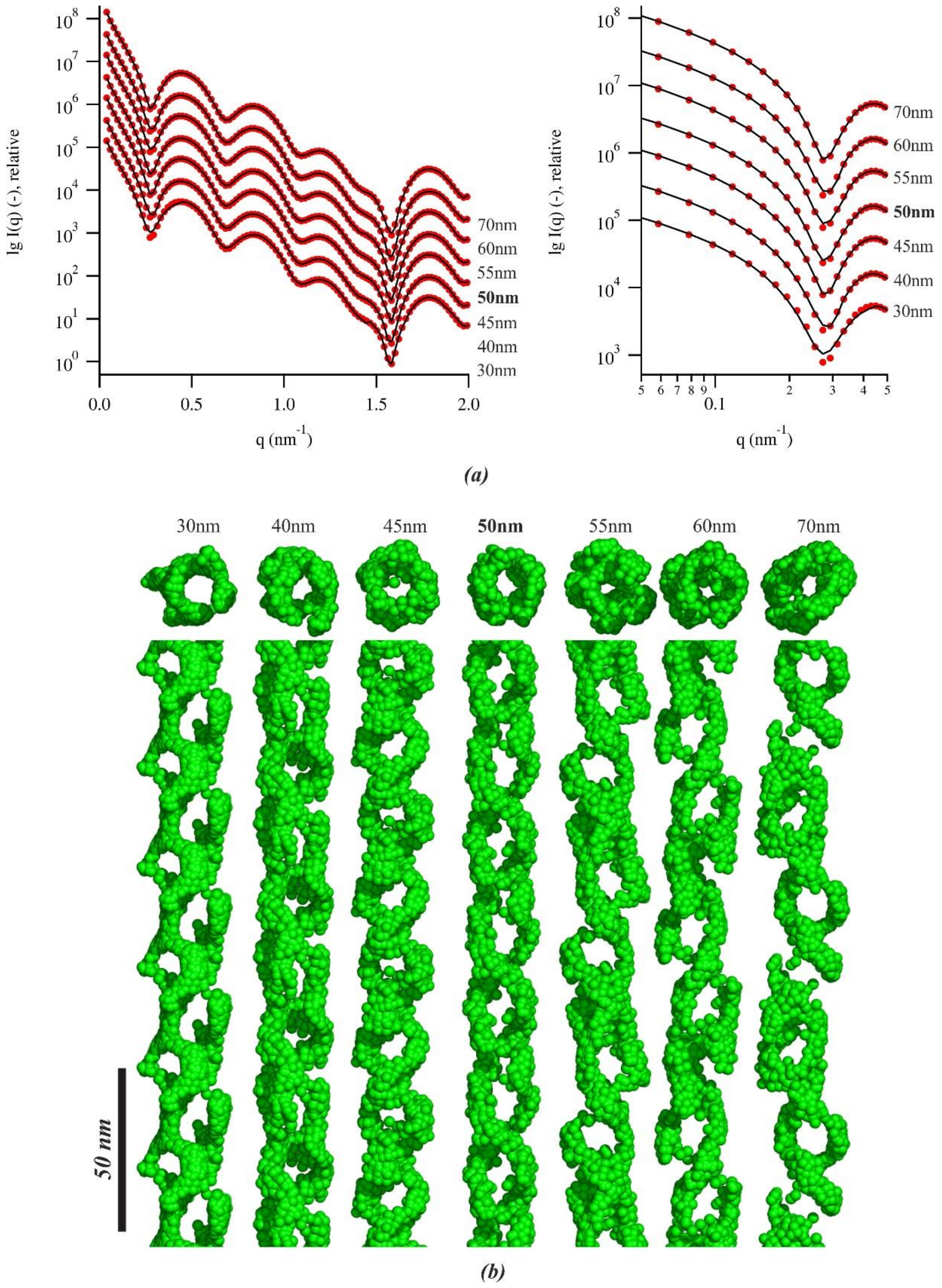

Figure S9 Reconstruction of model ***F*** (see Table 2 of the main text) with a building-block height of $50nm$ using a series of wrong building-block stack distances H_{BB}. The fitted curves are shown in (a): here, none of the fits show deviations from the model scattering-pattern even in in the low-q regime (see inset). In agreement with the investigation made on model ***D*** (see Supporting Figures S7 & S87) the corresponding reconstructions shown in (b) present artefacts if H_{BB} deviates by more than 10% from the real value. Point-representations of the reconstructions can be found in Supporting Figure S10.

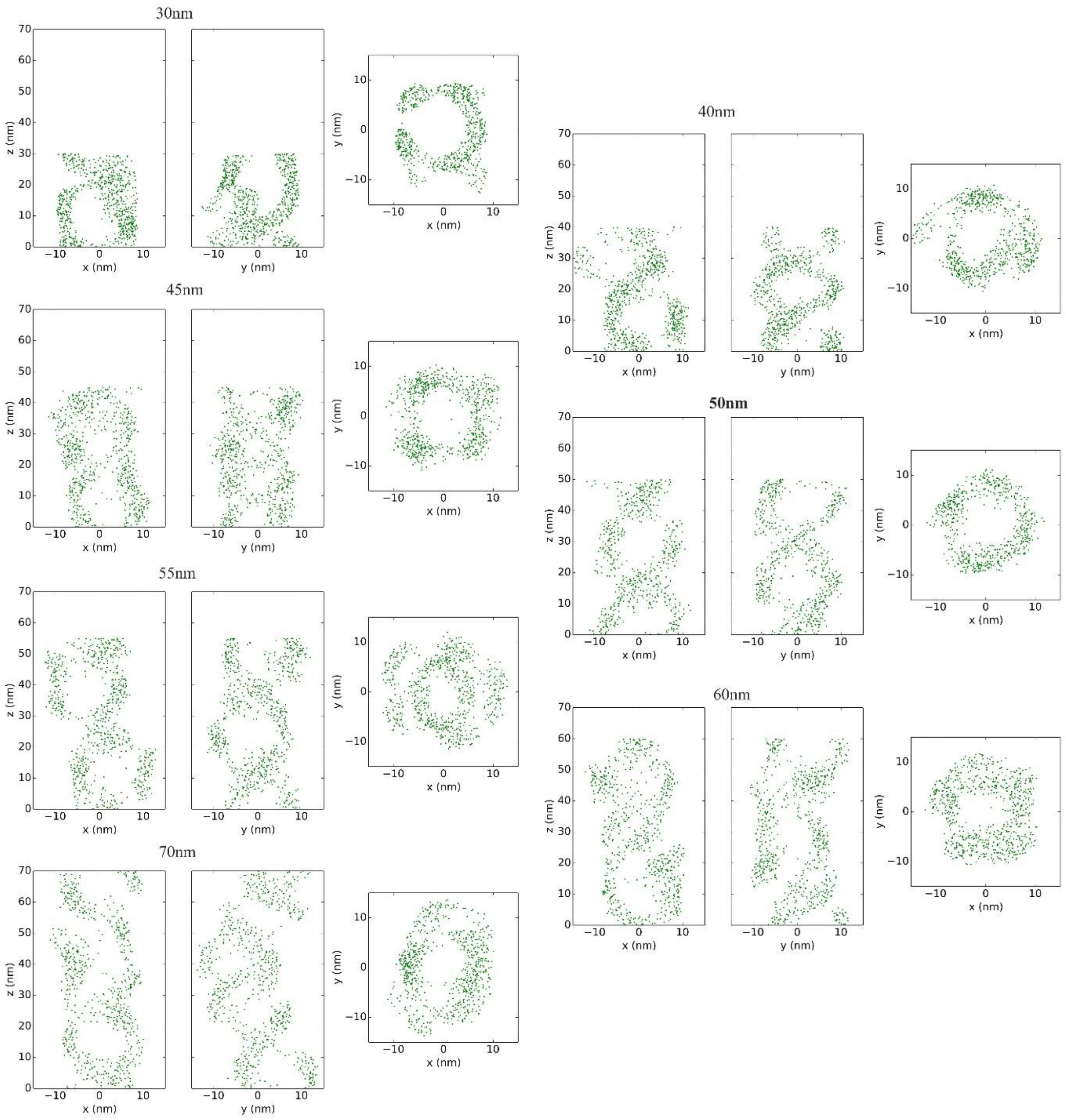

Figure S10 Point-representations corresponding to Supporting Figure S9, showing orthogonal views of the reconstructions of model $\boldsymbol{F}$ using a wrongly chosen stacking distance H_{BB}.

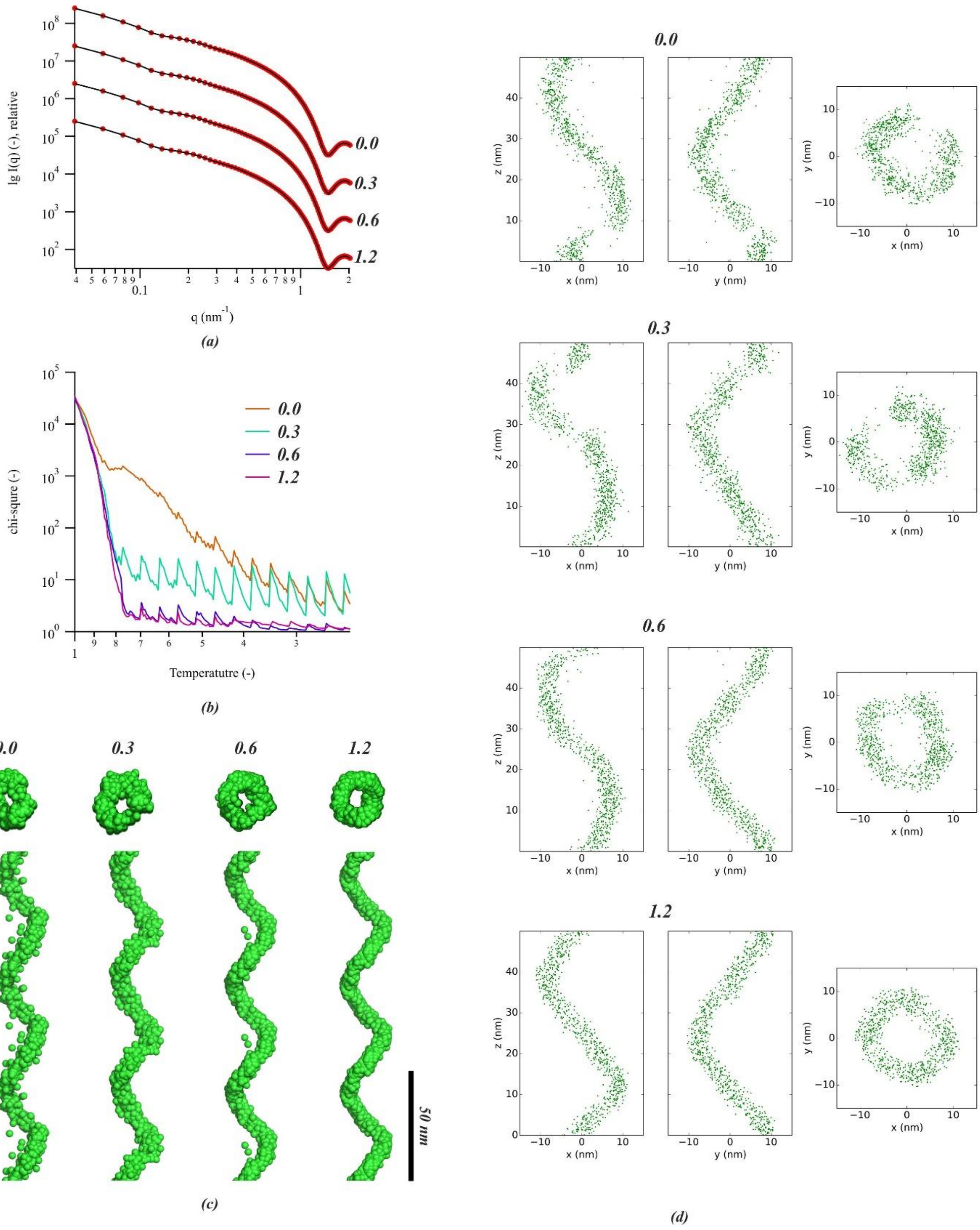

Figure S11 Reconstructions of model ***D*** (see Table 2 of the main text) using a variable helical bias parameter γ. (a) Scattering curves of the analytical model and the corresponding fits. The annotations ***0.0***, ***0.3, 0.6*** and ***1.2*** denote the actual γ value used for the reconstruction (0.3 is the default value for all other reconstructions shown in this work). (c) Convergence of the fitting algorithm shown by the decrease of the chi-square functional (see equation 3 of the main text) as a function of the annealing temperature. (c) Resulting reconstructions according to the scattering curves in (a). (d) Point representations of the corresponding models, showing orthogonal views of the reconstructions.

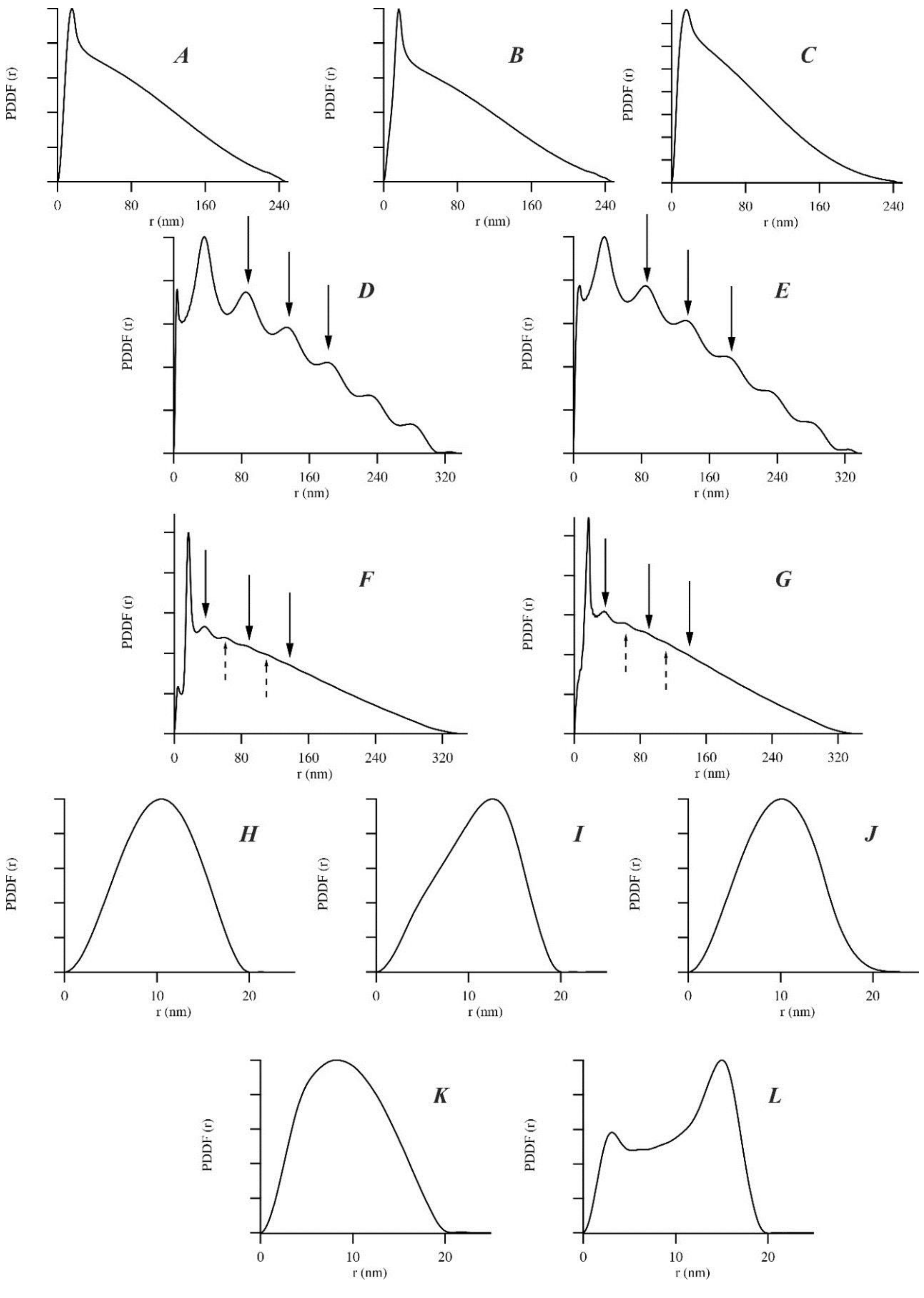

Figure S12 Pair distance distribution functions (PDDF) obtained from the scattering intensities presented in Figures 2 and 4. All curves were fitted from the analytical data using the GIFT software package (Bergmann *et al.*, 2000). The black arrows indicate the peaks linked to the recurrence of the building-block motif (see Supporting Information section S1 for details).

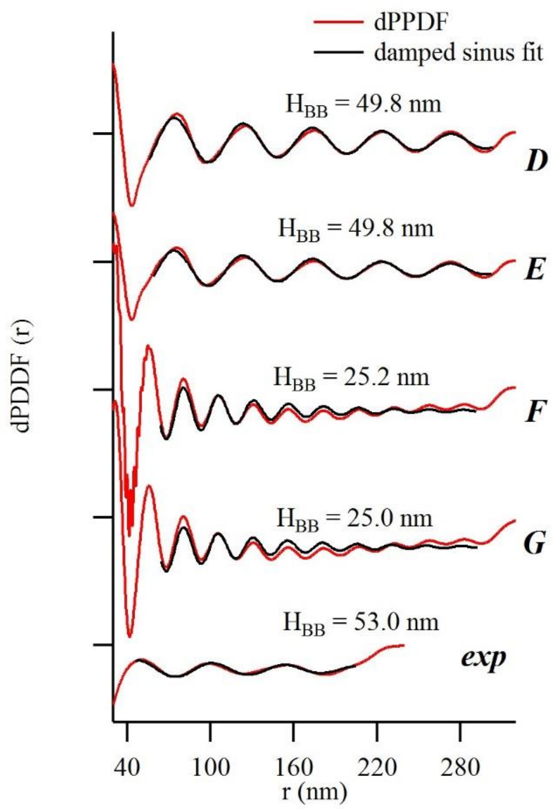

Figure S13 dPDDFs of the helical models (***A***-***G***) as well as of the experimental data set (***exp***) shown in Figure 2 and Figure 5 of the main text, respectively. The derivatives were calculated numerically from the PDDFs in Supporting Figure S11 and in inset of Figure 5. The dPDDFs were then fitted using the damped sine function according to equation S1. The resulting H_{BB} parameter is specified for each fit. Obviously, the determined stacking-distance of the double-strand models (***F*** and ***G***) is half of the real building-block size. This is due to the symmetry within the building-block: in a symmetric double helix, a building-block of pitch P with a full turn of 2π is equal to two stacked-units of turn π and pitch $P/2$.

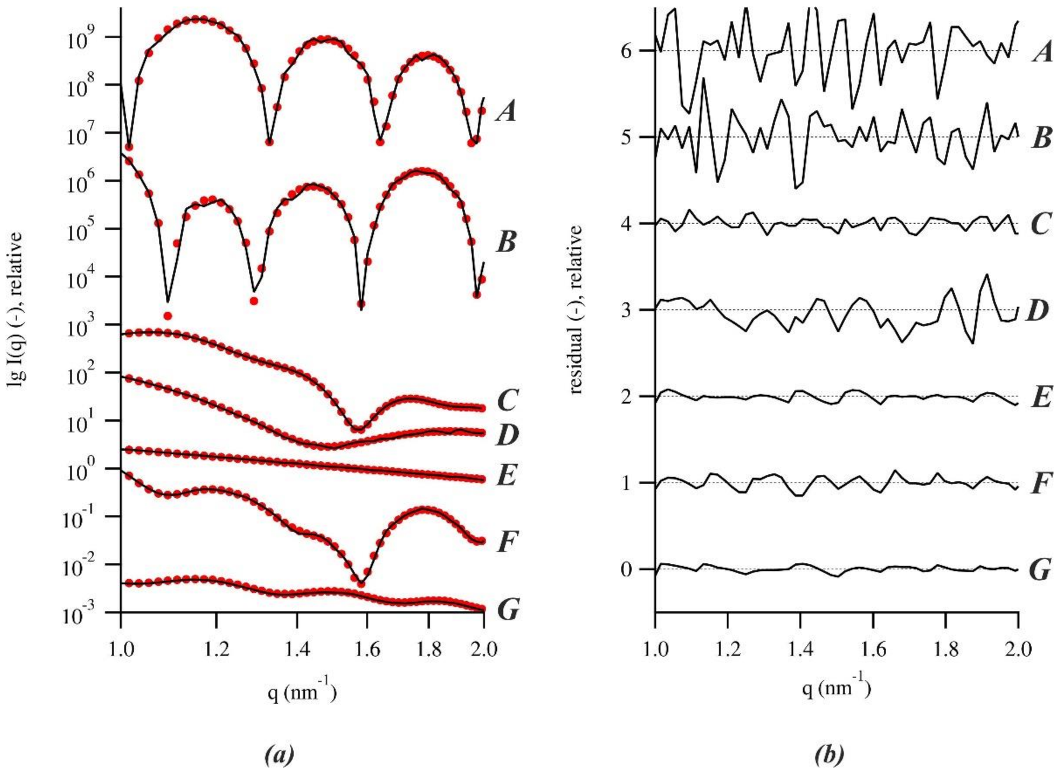

Figure S14 (a) Magnification of the high-q regime of the scattering functions computed from the seemingly endless geometries in Figure 2. In this magnified representation, slight oscillations of the projection scheme patterns are visible. (b) Residual curves of the corresponding fits.

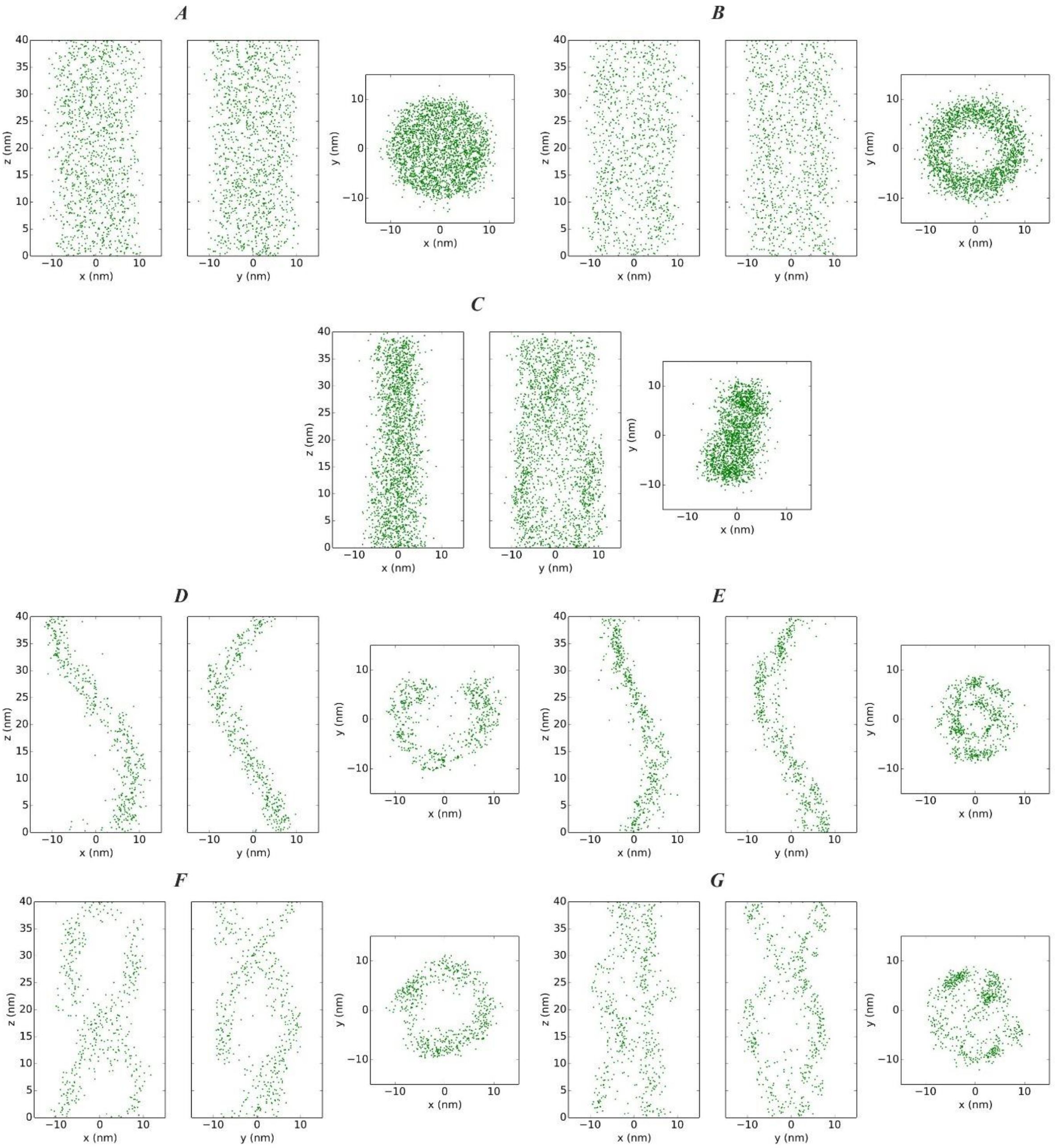

Figure S15 Point representations corresponding to Figure 3 of the main text, showing orthogonal views of the reconstructions from models ***A-G*** (see Table 2 of the main text).

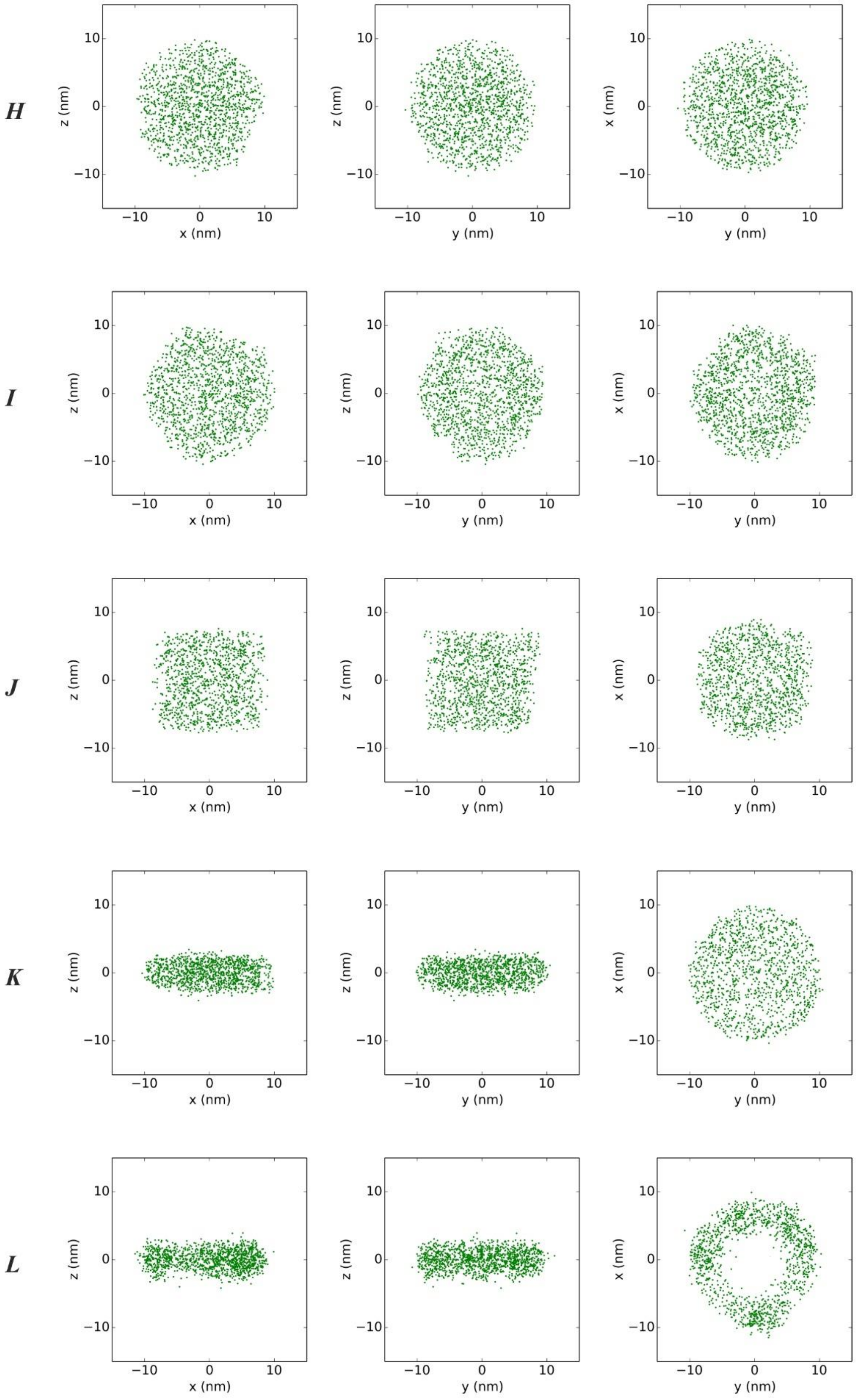

Figure S16 Point representations corresponding to Figure 4 of the main text, showing orthogonal views of the reconstructions from models ***H-L*** (see Table 2 of the main text).

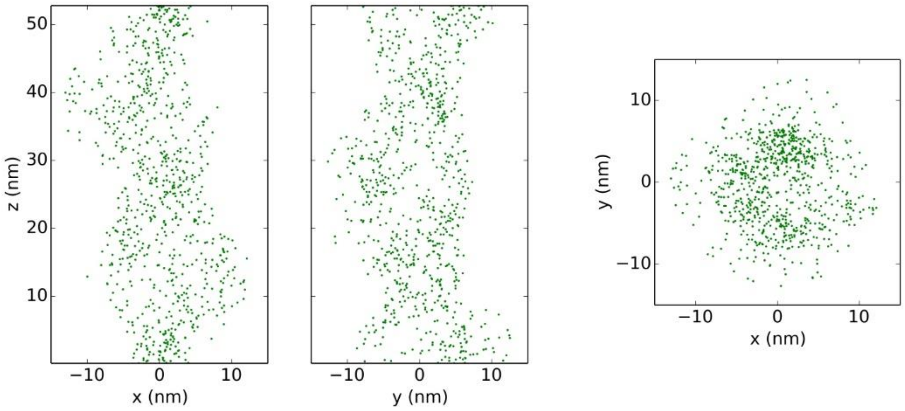

(a)

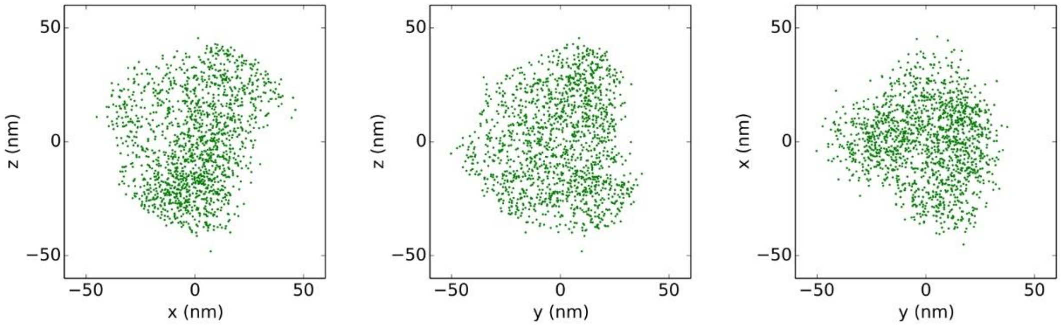

(b)

Figure S17 Point representations corresponding to Figure 5 of the main text, showing orthogonal views of the reconstructions from experimental data of the (a) elongated and (b) globular case.

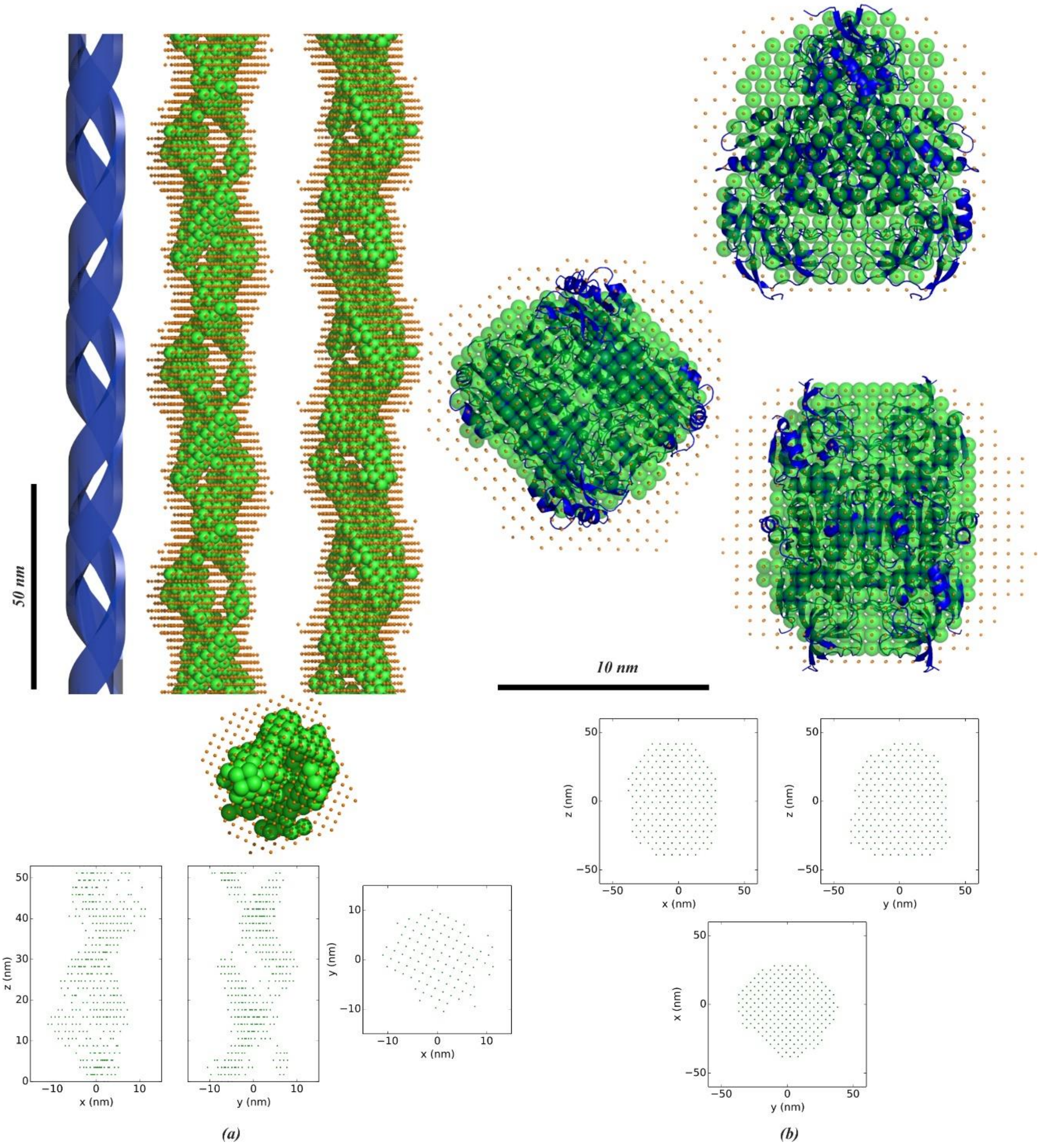

Figure S18 Analysis of uniqueness and repeatability of the reconstruction from experimental scattering data of the (a) elongated and (b) globular case. In each case, 16 separate reconstructions were performed and subsequently averaged by DAMAVER (Volkov & Svergun, 2003). The dots denote positions of the full DAMAVER model whereas the green spheres correspond to the 50% occupancy representation (see orthogonal views at the bottom). The theoretical models are shown in blue.

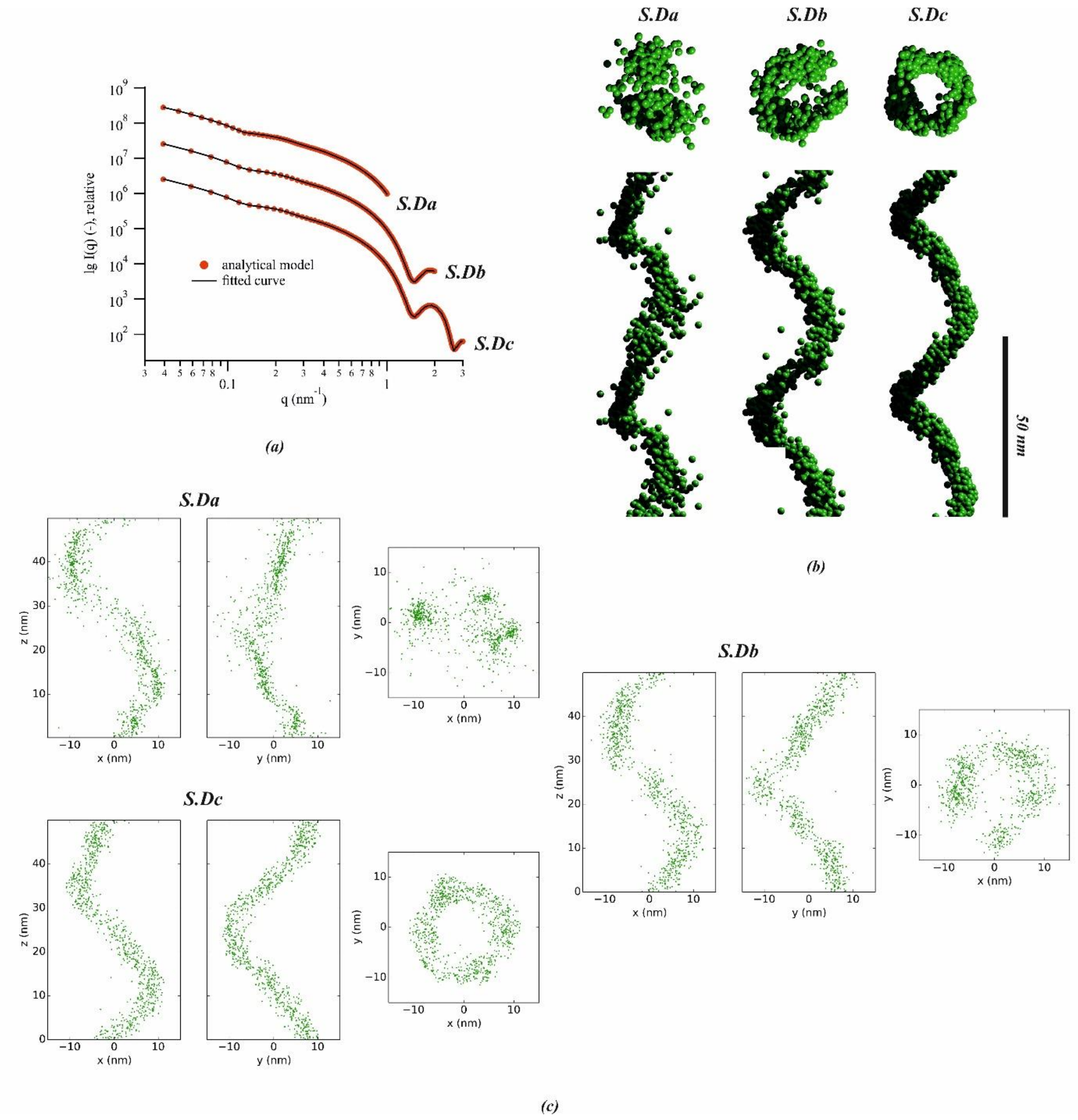

Figure S19 Reconstructions of model ***D*** (see Table 2 of the main text) using a variable angular range. (a) Truncated scattering curves of the analytical model and the corresponding fits. The curves ***S.Da***, ***S.Db*** and ***S.Dc*** correspond to a real-space resolution of 3.1, 1.6 and 1.0 nm, respectively. (b) Resulting reconstructions according to the scattering curves above. Obviously, the helical motif is still visible in all three cases, yet the cross-section can only reliably be restored in the latter two cases. (c) Point representations of the corresponding models, showing orthogonal views of the reconstructions.

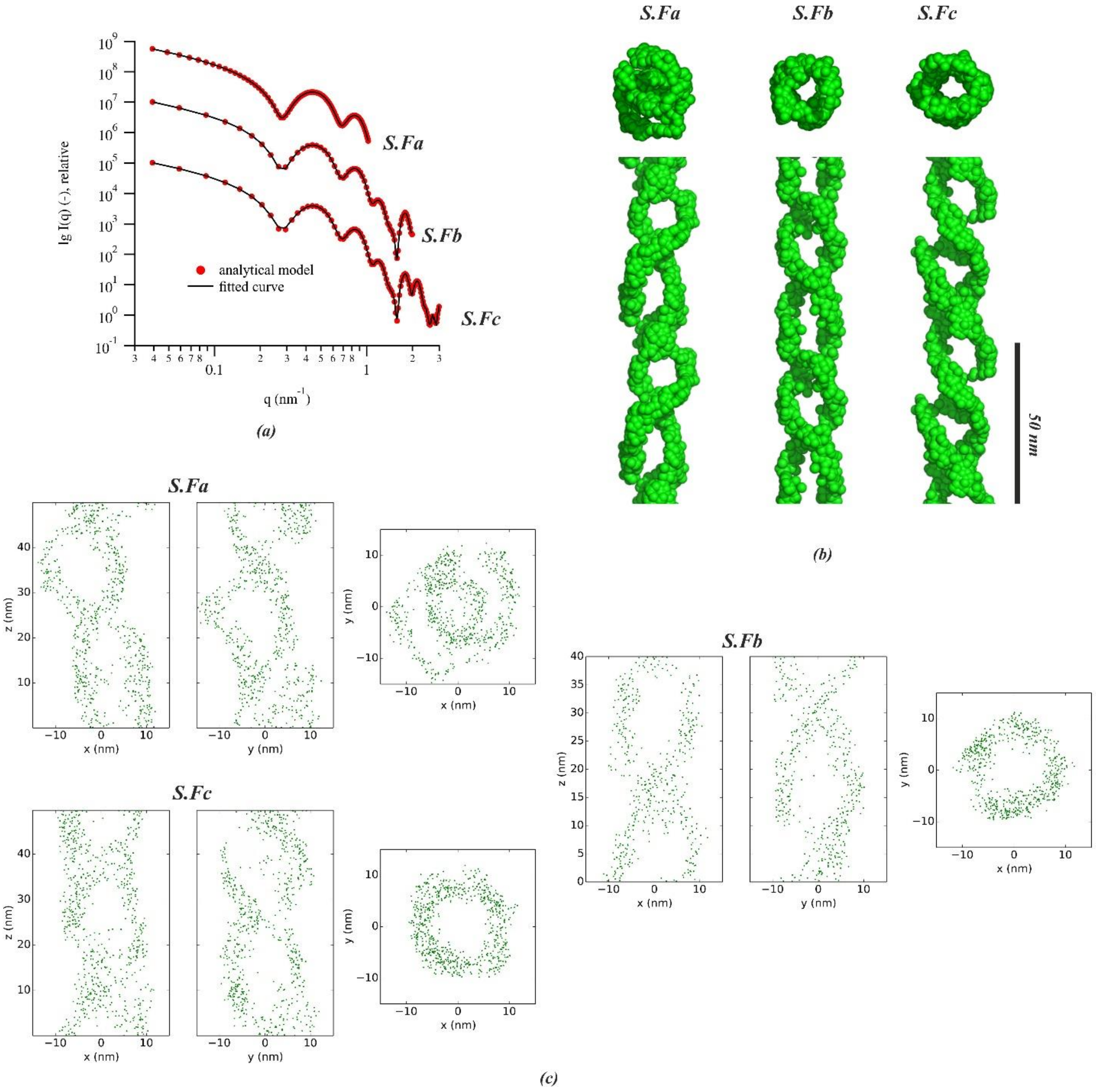

Figure S20 Reconstructions of model ***F*** (see Table 2 of the main text) using a variable angular range. (a) Truncated scattering curves of the analytical model and the corresponding fits. The curves ***S.Fa***, ***S.Fb*** and ***S.Fc*** correspond to a real-space resolution of 3.1, 1.6 and 1.0 nm, respectively. (b) Resulting reconstructions according to the scattering curves above. Here, the helical double-strand motif is clearly visible in all three cases. At lower resolution, inhomogeneities in the DA density are witnessed. Increasing the q-range and thus the resolution, the DA distribution becomes more homogenous. (c) Point representations of the corresponding models, showing orthogonal views of the reconstructions.

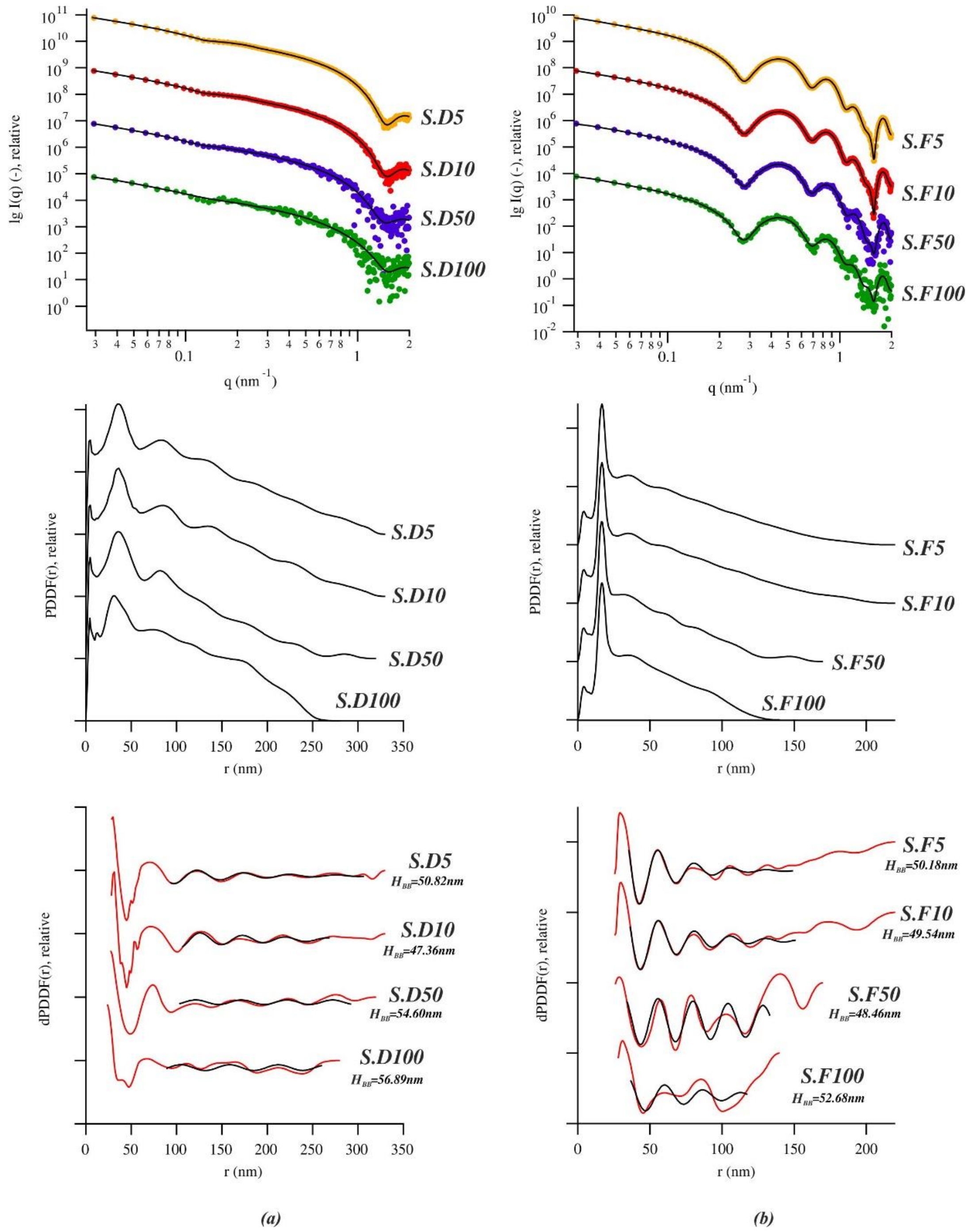

Figure S21 Influence of increasing noise level on the determined stacking distance. (Top) Scattering curves of (a) single strand helices and (b) double strand helices (see model ***D*** and ***F*** in Table 2 of the main text, respectively) with varying noise level (colored dots) together with the PDDF fits (black lines). The specified number denotes the scalar $E\ [nm^{-\frac{1}{2}}]$ used to calculate the noise level according to $E * \sqrt{I(q)}$. Hence, at $q_{max} = 2\ \mathrm{nm}^{-1}$, the relative noise compared to the scattering signal is approx. 15, 30, 150 and 300 % for E = 5, 10, 50 and 100, respectively. (Middle): PDDFs of the corresponding scattering curves. (Bottom) Derivatives of the PDDFs (dPDDFs – red lines) as well as the fitted sinusoidal functions from which the stacking distances are determined (black lines).

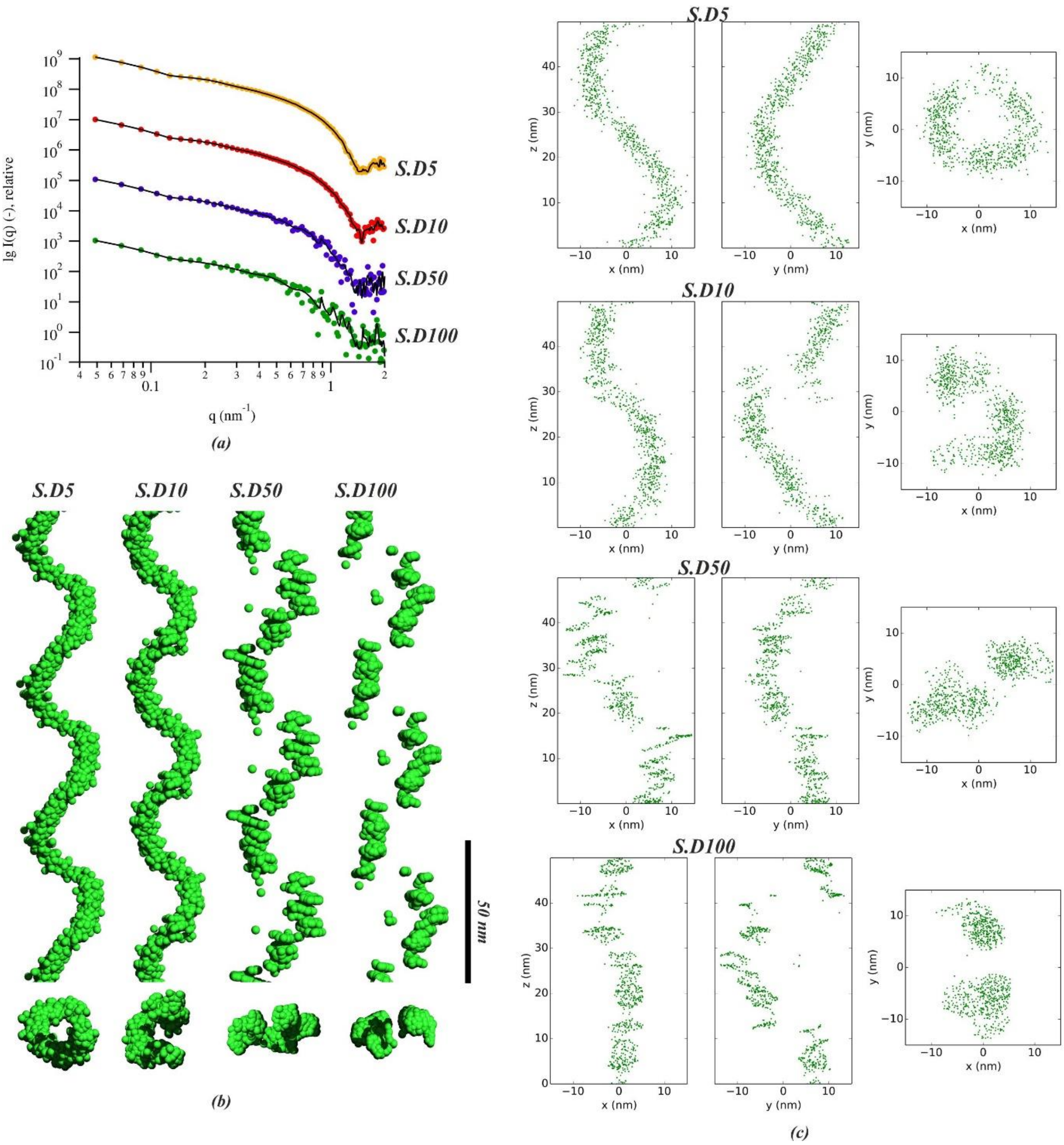

Figure S22 Reconstructions from the scattering patterns of model ***D*** (see Table 2 of the main text) using an increasing artificial error band. (a) Model scattering curves together with the corresponding fits. At $q_{max} = 2\ \mathrm{nm}^{-1}$, the relative noise compared to the scattering signal is approx. 15, 30, 150 and 300 % for ***S.D5, S.D10, S.D50*** and ***S.D100***, respectively. (b) Resulting reconstructions according to the scattering curves above. The helical motif is visible in cases ***S.D5*** and ***S.D10*** whereas for higher noise levels DA clustering is observed. (c) Point representations of the corresponding models, showing orthogonal views of the reconstructions.

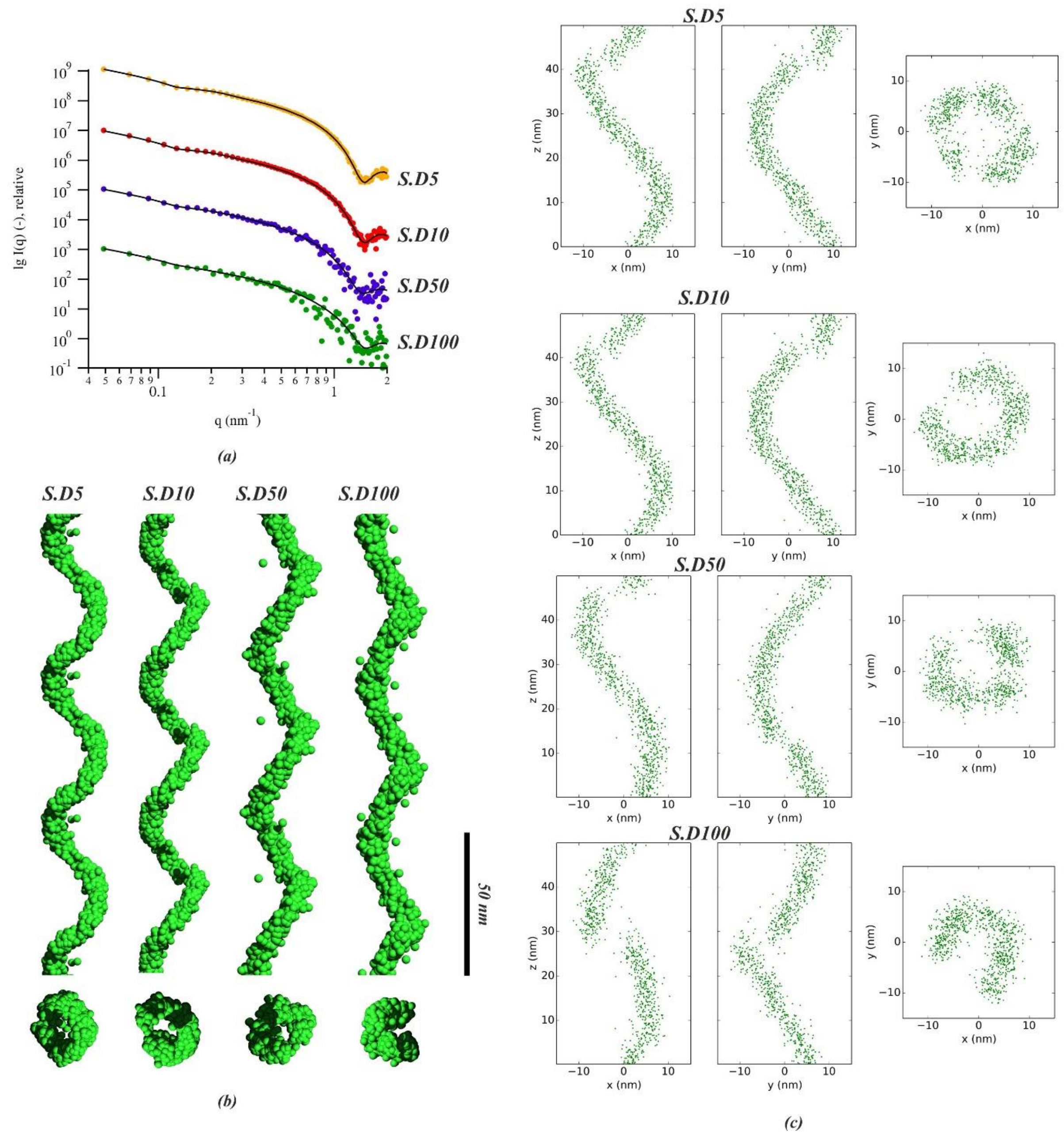

Figure S23 Reconstructions from scattering patterns *of the fitted PDDF* curves from model ***D*** (see Table 2 of the main text) using an increasing artificial error band. (a) Model scattering curves together with the corresponding fits. At $q_{max} = 2\ \mathrm{nm}^{-1}$, the relative noise compared to the scattering signal is approx. 15, 30, 150 and 300 % for ***S.D5, S.D10, S.D50*** and ***S.D100***, respectively. (b) Resulting reconstructions according to the scattering curves above. In all of the cases, the helical motif is clearly present and no DA clustering is observed. (c) Point representations of the corresponding models, showing orthogonal views of the reconstructions.

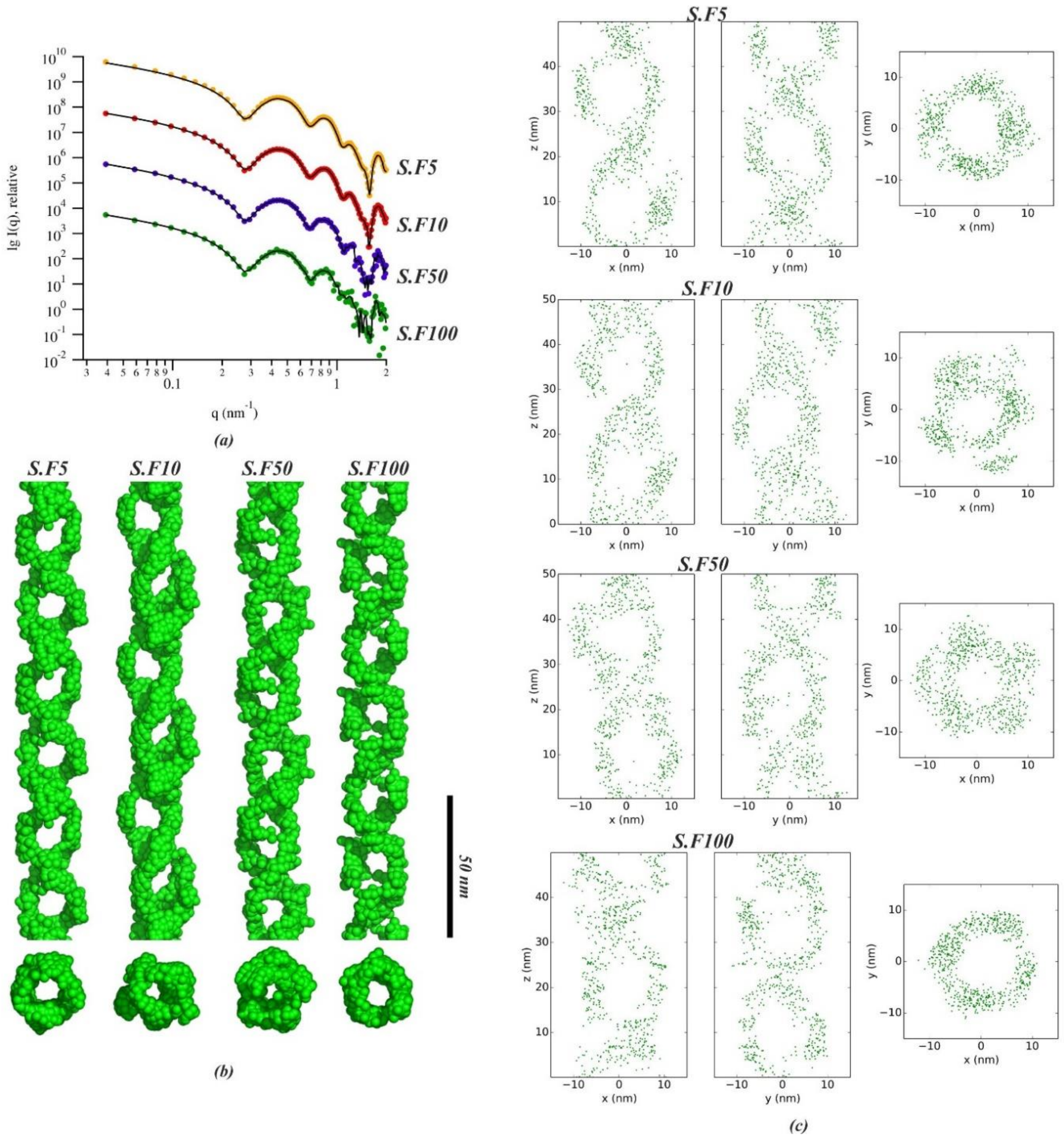

Figure S24 Reconstructions from the scattering patterns of model ***F*** (see Table 2 of the main text) using an increasing artificial error band. (a) Model scattering curves together with the corresponding fits. At $q_{max} = 2\ \mathrm{nm}^{-1}$, the relative noise compared to the scattering signal is approx. 15, 30, 150 and 300 % for ***S.F5, S.F10, SF50*** and ***S.F100***, respectively. (b) Resulting reconstructions according to the scattering curves above. The double stranded helical motive is visible in all cases. At higher noise levels, (***SF50*** and ***S.F100***) the strand surface becomes increasingly distorted and single, free floating DAs can be observed. (c) Point representations of the corresponding models, showing orthogonal views of the reconstructions.

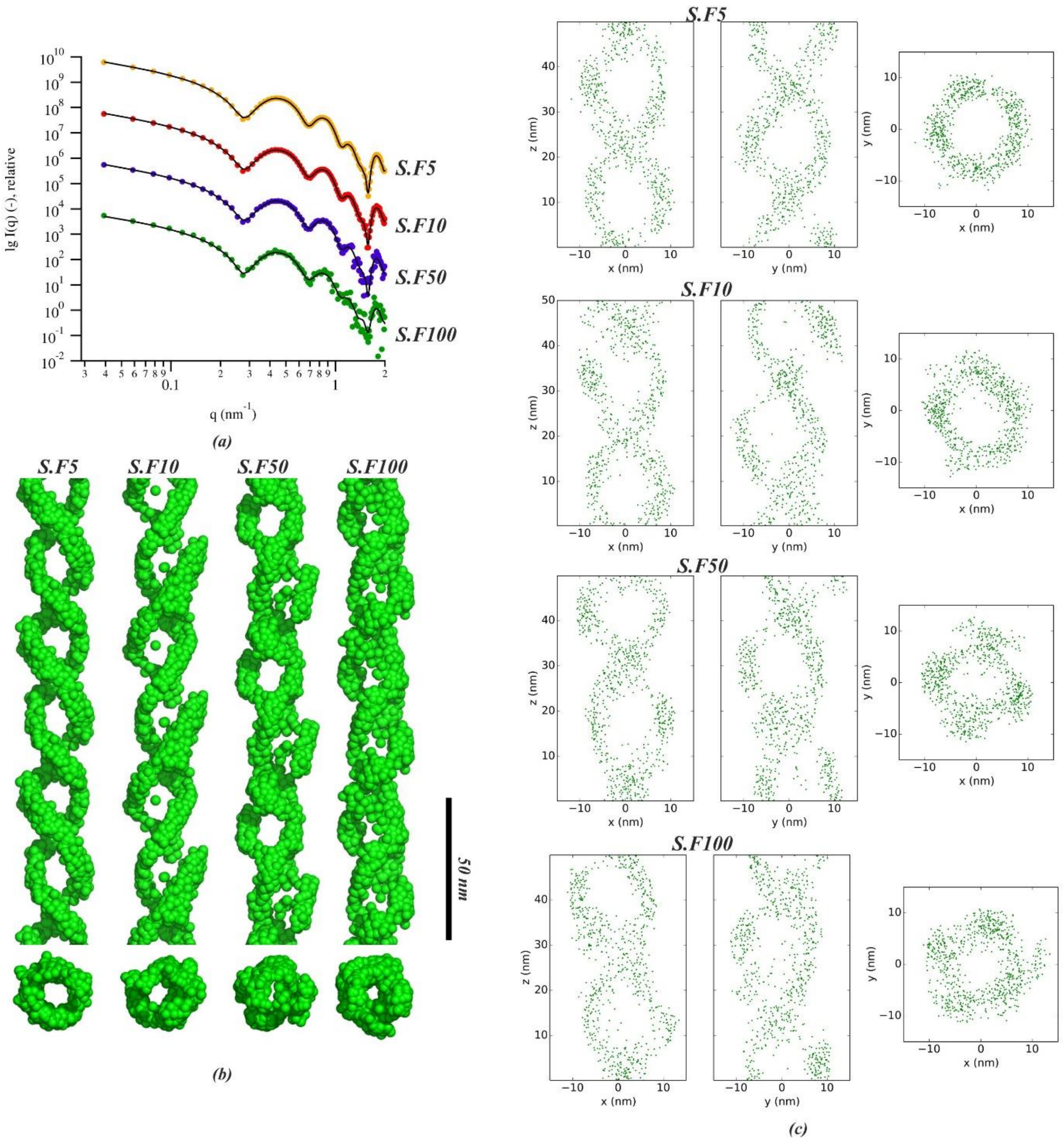

Figure S25 Reconstructions from scattering patterns *of the fitted PDDF* curves from model F (see Table 2 of the main text) using an increasing artificial error band. (a) Model scattering curves together with the corresponding fits. At $q_{max} = 2\ \mathrm{nm}^{-1}$, the relative noise compared to the scattering signal is approx. 15, 30, 150 and 300 % for ***S.F5, S.F10, S.F50*** and ***S.F100***, respectively. (b) Resulting reconstructions according to the scattering curves above. Same as in Supporting Figure S22, the double stranded helical motif is clearly present in all cases. (c) Point representations of the corresponding models, showing orthogonal views of the reconstructions.

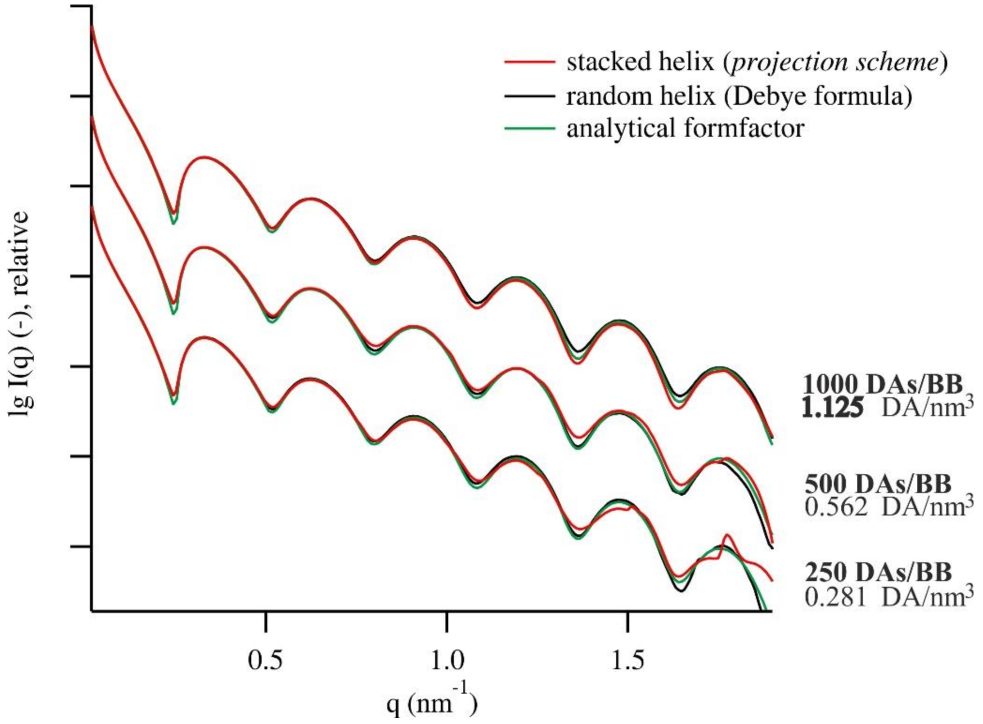

Figure S26 Scattering curves of an artificially filled single-strand helix with varying numbers of dummy atoms (DAs) per building block – stacked (red) and random (black) - in comparison to its analytical form factor (green). Obviously, the agreement between the artificial scattering curves (red and black) and the analytical model (green) becomes better when the number of DAs increases. However, the scattering data calculated using the proposed projection scheme (red) presents slight resonance effects in the upper q-regime. In congruence to above, this effect decreases when the DA density rises. (Details see Supporting Information section S2.3)

```
for k = 1 to N_k { // algorithm consists of N_k iterations
        T = T_0 * q_T^(k-1) // set annealing temperature
        randomly mix DA sequence in X_0

        for i = 1 to N_BB { // fitting procedure over all DAs
                X_new = X_0 where r_i,new = r_i,0 + r_rand(T) // random movement of i^th DA
                if ( f(X_new ) < f(X_0) // check for improvement of f(X) and
                        X_0 = X_new // accept movement
        }       } // all DAs have been considered

        if (mod(k, 2) == 0 { // every 2nd run of k
                for i = 0 to N_BB { // check all DAs
                        if (|r_i| > 2 * D_X0) { // if DAs are outside the critical radius
                                r_i *= 1/3 //move DA towards COM
        }       }       } // all DAs have been considered

        if (mod(k, 10) == 0 { // every 10th run of k
                for i = 0 to N_BB { // check all Das
                        if (D_12,i > 2 * ⟨D_12,X0⟩ and |r_i| > ⟨|r|⟩_X) { // if DAs are free floating
                                r_i += 1/2 * r_NN,i(N_BB/8) // move DAs towards N_BB/8 next neighbors
                        }
                        r_i,new = r_i,0 + r_rand(T) // force random movement of i^th DA
}       }       } // end of all k iterations
```

Figure S27 Pseudo-code implementation of the algorithm implemented in SasHel. For description of variables and further details see Supporting Information section S3.
